# Single-base resolution of mouse offspring brain methylome reveals epigenome modifications caused by gestational folic acid

**DOI:** 10.1186/1756-8935-7-3

**Published:** 2014-02-03

**Authors:** Subit Barua, Salomon Kuizon, Kathryn K Chadman, Michael J Flory, W Ted Brown, Mohammed A Junaid

**Affiliations:** 1 Department of Developmental Biochemistry, New York State Institute for Basic Research in Developmental Disabilities, 1050 Forest Hill Road, Staten Island, NY 10314, USA; 2 Department of Developmental Neurobiology, New York State Institute for Basic Research in Developmental Disabilities, 1050 Forest Hill Road, Staten Island, NY 10314, USA; 3 Department of Infant Development, New York State Institute for Basic Research in Developmental Disabilities, 1050 Forest Hill Road, Staten Island, NY 10314, USA; 4 Department of Human Genetics, New York State Institute for Basic Research in Developmental Disabilities, 1050 Forest Hill Road, Staten Island, NY 10314, USA; 5 Structural Neurobiology Laboratory, Department of Developmental Biochemistry, New York State Institute for Basic Research in Developmental Disabilities, 1050 Forest Hill Road, Staten Island, NY 10314, USA

## Abstract

**Background:**

Epigenetic modifications, such as cytosine methylation in CpG-rich regions, regulate multiple functions in mammalian development. Maternal nutrients affecting one-carbon metabolism during gestation can exert long-term effects on the health of the progeny. Using C57BL/6 J mice, we investigated whether the amount of ingested maternal folic acid (FA) during gestation impacted DNA methylation in the offspring’s cerebral hemispheres. Reduced representation bisulfite sequencing at single-base resolution was performed to analyze genome-wide DNA methylation profiles.

**Results:**

We identified widespread differences in the methylation patterns of CpG and non-CpG sites of key developmental genes, including imprinted and candidate autism susceptibility genes (*P* <0.05). Such differential methylation of the CpG and non-CpG sites may use different mechanisms to alter gene expressions. Quantitative real time reverse transcription-polymerase chain reaction confirmed altered expression of several genes.

**Conclusions:**

These finding demonstrate that high maternal FA during gestation induces substantial alteration in methylation pattern and gene expression of several genes in the cerebral hemispheres of the offspring, and such changes may influence the overall development. Our findings provide a foundation for future studies to explore the influence of gestational FA on genetic/epigenetic susceptibility to altered development and disease in offspring.

## Background

The folate cycle, in conjunction with one-carbon metabolism, facilitates nucleic acid synthesis and is responsible for the transfer of 1-carbon methyl groups to DNA and proteins. Methyl groups added onto cytosine residues in promoter region CpGs in genomic DNA are central to the regulation of gene expression [1,2]. The role of folic acid (FA) in preventing neurodevelopmental disorders and birth defects has long been recognized, and as such, its use during pregnancy is strongly emphasized [3-5]. Dietary FA supplementation is credited with a greater than 70% reduction in the incidence of neural tube defects (NTDs) in the US [6]. There has been speculation that FA supplementation may be associated with certain aberrant conditions in children [7-9], and a clear understanding of this purported association is essential in view of the presence of significant amounts of synthetic FA in our diets. Earlier, we reported that exposure of lymphoblastoid cells to FA supplementation causes widespread changes in gene expression [10]. We suggested that the occurrence of such epigenetic changes during gestational development may impact the methylation status of DNA in the offspring’s brain and cause altered gene expression. Because gestational development involves a highly orchestrated regulation of gene expression, such gene dysregulation may affect the development of the brain and may culminate in neuropsychiatric conditions. This could be a contributing factor to the increasing prevalence in recent years.

To test the hypothesis that excess FA supplementation could alter the methylation in the brains of offspring, 1 week prior to mating, a group of C57BL/6 J female mice were fed a custom AIN-93G amino acid-based diet (Research Diet, Inc., New Brunswick, NJ, USA), with FA at 0.4 mg/kg (low maternal folic acid, or LMFA) or 4 mg/kg (high maternal folic acid, or HMFA). FA at the 4 mg/kg level is above the range currently included in mice chow, whereas the 0.4 mg/kg level of FA has been found to be necessary for a normal healthy litter size [11]. FA at the 4 mg/kg level in mice roughly corresponds to the 4 mg/day dose in humans, which is the level of FA supplementation (4 mg/day) prescribed to women with a history of NTD pregnancy. We used an amino acid-based diet to precisely control the amount of FA in the diet. To understand the dynamics of DNA methylation, genomic DNA from the cerebral hemispheres of the offspring was isolated at postnatal day 1 segregated by gender, and high-resolution, single-base DNA methylation profiling was performed by using next-generation Illumina (Illumina Inc., San Diego, CA, USA) sequencing (details in the Methods section).

## Results

### Global DNA methylation patterns of the offspring’s cerebral hemispheres from high maternal folic acid

**Figure 1:**
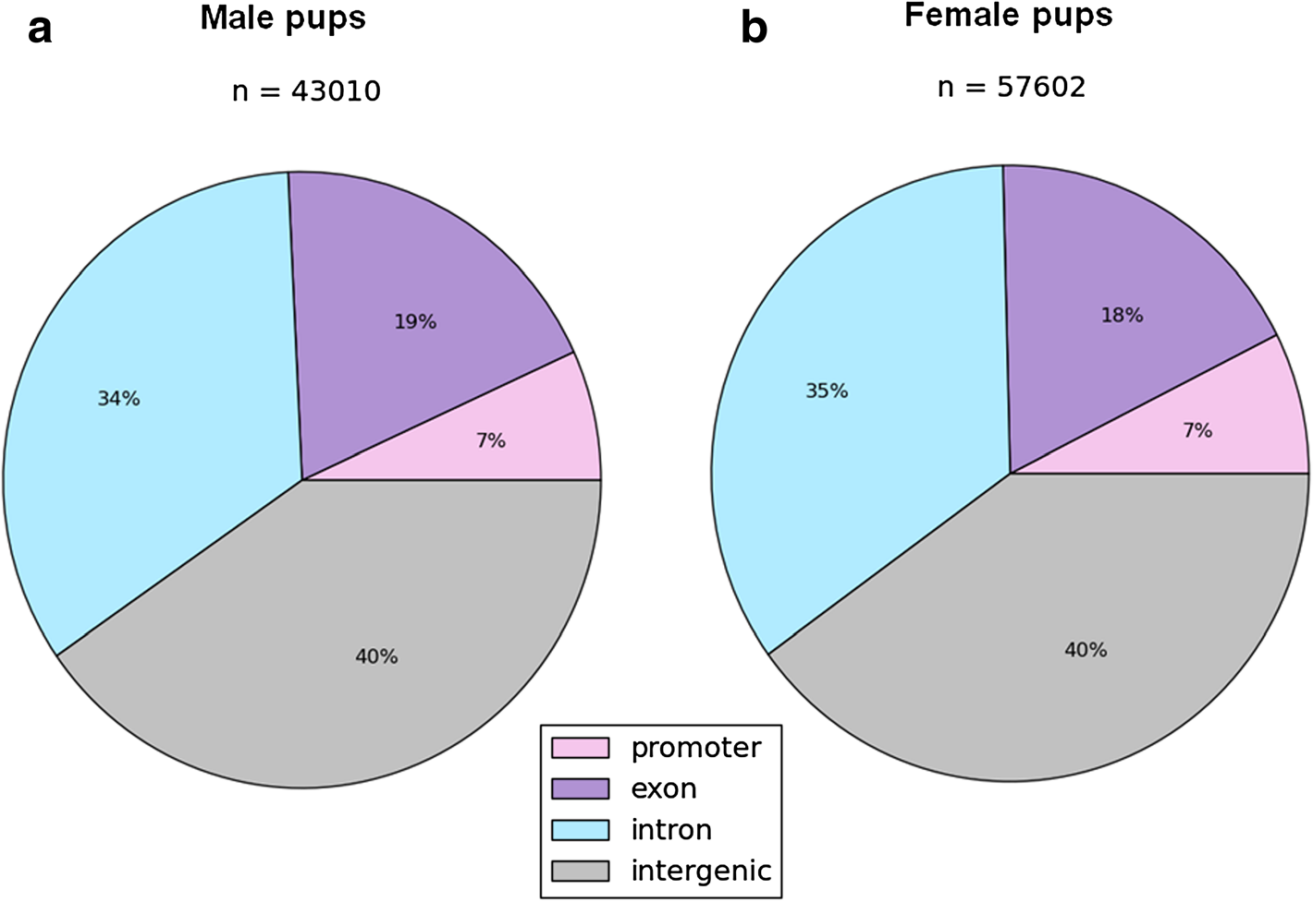
**Distribution of differentially methylated sites in CpG island sequences. (a)** Male low maternal folic acid (LMFA) versus high maternal folic acid (HMFA). **(b)** Female LMFA versus HMFA.

The final DNA methylation map presented in this study represents the summary of three biological replicates [12,13], with each mouse collected from an independent litter. On average, the sequence depths of unique CpG sites in our study were 4,647,138 (11 times) for male and 4,410,480 (14 times) for female DNA samples (Additional file 1: Table S1), and about 90% of the CpG islands were covered. To investigate the differentially methylated regions (DMRs), sequence alignment and Fisher’s exact test or *t* test were performed for each CpG site that had at least five reads covered. Results of global methylation comparison revealed that approximately 16% of the CpG sites were differentially methylated in both male and female pups from HMFA (n = 43,010 for male, n = 57,602 for female). The majority of the CpG island-associated DMRs were either intergenic or in introns, whereas 18% to 19% were in exons, and approximately 7% were in promoter regions (Figure 1a, b). Several genes involved in neural functioning, brain development, and synaptic plasticity were differentially methylated (*P* <0.05) in the CpG sites of the offspring from HMFA (Tables 1, 2, 3 and 4, Additional file 2: Table S2, Additional file 3: Table S3, Additional file 4: Table S5, and Additional file 5: Table S6). The results of high-resolution global DNA methylation profiling indicated that maternal FA induces significant changes in the overall methylation patterns in the brains of the offspring. The correlations of the distribution of methylation ratios in male and female pups for the corresponding sites in LMFA and HMFA are shown in Additional file 6: Figures S1-S6, and the distributions of the overlapped sites between LMFA and HMFA male with that of LMFA and HMFA female differential methylation sites (*P* <0.05) are shown as a hexbin plot in Additional file 7: Figures S7-S9.

**Table 1:** List of hypermethylated CpG sites in the promoter of genes from high maternal folic acid diet

**Chromosome**	**Start**	**End**	**Gene**	**Total CpG LMFA**	**Total CpG HMFA**	**Methylation difference**	***P value***
**Male-CpG**							
Chr1	174430052	174430053	*Tagln2*	12	5	-0.80	0.00
Chr2	124976701	124976702	*Slc12a1*	9	10	-0.79	0.00
Chr2	163576501	163576502	*Ada*	7	5	-0.80	0.01
Chr5	110658438	110658439	*Ankle2*	10	6	-0.80	0.01
Chr7^a^	25654980	25654981	*Dmrtc2*	5	5	-1.00	0.01
Chr12^a^	112951515	112951516	*Bag5*	20	5	-0.80	0.00
Chr13	21533599	21533600	*Pgbd1*	11	5	-0.91	0.00
Chr14	12384528	12384529	*Ptprg*	15	6	-0.87	0.00
Chr16	4078495	4078496	*Trap1*	17	20	-0.85	0.00
Chr18^a^	25327450	25327451	*AW554918*	11	10	-0.80	0.00
Chr18	55150289	55150290	*Zfp608*	8	5	-0.80	0.01
ChrX	70963189	70963190	*Bcap31*	15	7	-0.80	0.00
**Female-CpG**							
Chr1	158239364	158239365	*Nphs2*	5	5	-0.80	0.05
Chr2	30141110	30141111	*Nup188*	10	9	-0.78	0.00
Chr2	93663150	93663151	*Ext2*	7	9	-0.78	0.00
Chr4	115456244	115456245	*Atpaf1*	10	5	-0.80	0.01
Chr7	4765788	4765789	*Ube2s*	12	14	-0.79	0.00
Chr7	116076508	116076509	*Eif3f*	8	10	-0.80	0.00
Chr9	43921237	43921238	*Rnf26*	10	6	-0.80	0.01
Chr9	43921243	43921244	*Rnf26*	10	6	-0.80	0.01
Chr9	70352700	70352701	*Rnf111*	7	6	-1.00	0.00
Chr11^a^	106640875	106640876	*Polg2*	8	8	-0.75	0.01
Chr12^a^	111517321	111517322	*Dio3*	8	8	-0.75	0.01
Chr13	3147545	3147546	*Speer6-ps1*	6	5	-0.80	0.02
Chr13	38751553	38751554	*Eef1e1*	10	6	-0.80	0.01
Chr16	18624289	18624290	*Gp1bb*	8	5	-0.75	0.02
Chr17	24806277	24806278	*Zfp598*	7	6	-0.86	0.00
Chr18^a^	51277441	51277442	*Prr16*	7	5	-0.80	0.01
Chr18	60932782	60932783	*Rps14*	5	9	-0.78	0.02
ChrX	7721523	7721524	*2900002K06Rik*	6	13	-0.76	0.00
ChrX^a^	155852426	155852427	*Mtap7d2*	14	17	-0.82	0.00

^a^Methylation exclusively in the CpG island of promoter region. HMFA, high maternal folic acid; LMFA, low maternal folic acid.

**Table 2:** List of hypomethylated CpG sites in the promoter of genes from high maternal folic acid diet

**Chromosome**	**Start**	**End**	**Gene**	**Total CpG LMFA**	**Total CpG HMFA**	**Methylation difference**	***P value***
**Male-CpG**							
Chr2	104335383	104335384	*Hipk3*	6	10	-0.80	0.01
Chr2	118590048	118590049	*A430105I19Rik*	6	7	-0.83	0.00
Chr2	164156736	164156737	*Svs5*	5	6	-0.83	0.02
Chr2	171789984	171789985	*1700028P15Rik*	17	5	-0.80	0.00
Chr4	135283630	135283631	*Il22ra1*	5	5	-0.80	0.05
Chr5	122070856	122070857	*Acad12*	6	11	-0.73	0.01
Chr7	3219409	3219410	*Mir291b*	8	11	-0.75	0.00
Chr7	7253725	7253726	*Clcn4-2*	6	9	-0.72	0.01
Chr7^a^	15208940	15208941	*Gm18756*	10	12	-0.83	0.00
Chr7	91836603	91836604	*2610206C17Rik*	6	5	-0.83	0.02
Chr8	34495178	34495179	*Purg*	5	5	-0.80	0.05
Chr8	73034757	73034758	*Uba52*	6	6	-0.83	0.02
Chr8	77516944	77516945	*Hmgxb4*	6	15	-0.77	0.00
Chr9	66892554	66892555	*Tpm1*	15	6	-0.73	0.00
Chr10	53239171	53239172	*Gm20597*	8	7	-0.75	0.01
Chr12	3235150	3235151	*1700012B15Rik*	16	18	-0.88	0.00
Chr13	53382125	53382126	*Ror2*	6	5	-0.80	0.02
Chr13	53382128	53382129	*Ror2*	6	5	-1.00	0.00
Chr13	97839933	97839934	*Fam169a*	5	8	-0.75	0.02
Chr13	100671338	100671339	*Cartpt*	5	5	-0.80	0.05
Chr14	67628989	67628990	*Bnip3l*	7	8	-0.75	0.01
Chr19	5690281	5690282	*Pcnxl3*	20	13	-0.80	0.00
**Female-CpG**							
Chr2	127618583	127618584	*1500011K16Rik*	11	20	-0.85	0.00
Chr5^a^	100468191	100468192	*Enoph1*	11	5	-0.71	0.01
Chr6^a^	52196197	52196198	*Hoxa11*	5	5	-0.80	0.05
Chr6	100476908	100476909	*1700049E22Rik*	10	5	-0.80	0.00
Chr7	26326796	26326797	*Ceacam2*	6	15	-0.77	0.00
Chr7	29528469	29528470	*Mrps12*	16	5	-0.80	0.00
Chr7	86988105	86988106	*Anpep*	12	5	-0.83	0.00
Chr8	87469964	87469965	*Rtbdn*	7	6	-0.86	0.00
Chr8	116657191	116657192	*Nudt7*	14	5	-0.71	0.01
Chr9	109833746	109833747	*Mtap4*	8	14	-0.71	0.00
Chr10	76992742	76992743	*Itgb2*	8	7	-0.71	0.01
Chr10	80846466	80846467	*Dohh*	8	13	-0.69	0.00
Chr11	88727116	88727117	*Akap1*	6	5	-0.80	0.02
Chr11	115184397	115184398	*Ush1g*	12	5	-0.75	0.01
Chr11	118204319	118204320	*BC100451*	20	7	-0.81	0.00
Chr11	119909421	119909422	*Aatk*	7	5	-0.71	0.03
Chr11	120051942	120051943	*2810410L24Rik*	10	5	-0.70	0.02
Chr12	52447904	52447905	*G2e3*	8	5	-0.75	0.02
Chr14^a^	63380523	63380524	*Ints6*	5	6	-0.80	0.02
Chr15	81561248	81561249	*Rangap1*	13	5	-0.72	0.01
Chr17	52020946	52020947	*Gm20098*	9	7	-0.71	0.00
Chr18	38762241	38762242	*Spry4*	6	5	-0.83	0.02
Chr18	60933042	60933043	*Rps14*	9	8	-0.76	0.00
Chr19	7070128	7070129	*Trpt1*	8	6	-0.71	0.03

^a^Methylation exclusively in the CpG island of promoter region. HMFA, high maternal folic acid; LMFA, low maternal folic acid.

**Table 3:** List of top 20 hypermethylated CpG sites in the gene body of genes from high maternal folic acid diet

**Chromosome**	**Start**	**End**	**Gene**	**Total CpG LMFA**	**Total CpG HMFA**	**Methylation difference**	***P value***
**Male**							
Chr3	138455425	138455426	*Tspan5*	15	20	0.90	0.00
Chr4	119140980	119140981	*Rimkla*	21	15	0.79	0.00
Chr4	119140989	119140990	*Rimkla*	37	16	0.77	0.00
Chr4	119141016	119141017	*Rimkla*	21	15	0.79	0.00
Chr4	119610724	119610725	*Hivep3*	11	9	0.89	0.00
Chr8	87012542	87012543	*Cacna1a*	8	12	0.88	0.00
Chr8	94181294	94181295	*Fto*	28	22	0.79	0.00
Chr9	15678801	15678802	*Mtnr1b*	7	10	1.00	0.00
Chr9	106735686	106735687	*Vprbp*	12	12	1.00	0.00
Chr9	106735687	106735688	*Vprbp*	20	10	0.90	0.00
Chr9	110562402	110562403	*Ccdc12*	14	22	0.86	0.00
Chr10	115535492	115535493	*Ptprr*	14	9	0.86	0.00
Chr13	84421455	84421456	*Tmem161b*	8	12	0.88	0.00
Chr13	93030034	93030035	*Msh3*	363	63	0.83	0.00
Chr14	75232739	75232740	*Lrch1*	18	10	0.89	0.00
Chr15	89378341	89378342	*Shank3*	17	9	0.83	0.00
Chr18	37951652	37951653	*Pcdha4-g*	14	10	1.00	0.00
Chr18	60852504	60852505	*Ndst1*	12	6	1.00	0.00
Chr18	65119073	65119074	*Nedd4l*	18	17	0.89	0.00
Chr19	31290367	31290368	*Prkg1*	17	16	0.76	0.00
**Female**							
Chr2	25434932	25434933	*Gm996*	9	13	0.85	0.00
Chr3	30935498	30935499	*Prkci*	10	8	1.00	0.00
Chr3	103739555	103739556	*Rsbn1*	9	15	0.89	0.00
Chr4	126102080	126102081	*Eif2c3*	19	17	0.74	0.00
Chr4	140978449	140978450	*Hspb7*	6	19	0.83	0.00
Chr4	150546918	150546919	*Camta1*	22	6	0.83	0.00
Chr5	65200088	65200089	*Klf3*	47	36	0.72	0.00
Chr5	103970283	103970284	*Ptpn13*	23	12	0.74	0.00
Chr5	131698838	131698839	*Wbscr17*	21	16	0.73	0.00
Chr7	53799845	53799846	*Sergef*	14	21	0.71	0.00
Chr8	35200739	35200740	*Leprotl1*	25	10	0.76	0.00
Chr9	8001572	8001573	*Yap1*	11	15	0.73	0.00
Chr9	42341123	42341124	*Grik4*	39	30	0.73	0.00
Chr10	88898375	88898376	*Gas2l3*	14	11	0.82	0.00
Chr11	3211676	3211677	*Gm11944*	10	35	0.74	0.00
Chr11	115670841	115670842	*Caskin2*	8	14	0.86	0.00
Chr14	58310412	58310413	*Lats2*	6	24	1.00	0.00
Chr16	34322324	34322325	*Kalrn*	12	14	0.71	0.00
Chr17	86912790	86912791	*Prkce*	8	18	0.89	0.00
Chr19	25161873	25161874	*Dock8*	16	12	0.75	0.00

HMFA, high maternal folic acid; LMFA, low maternal folic acid.

**Table 4:** List of top 20 hypomethylated CpG sites in the genebody of genes from high maternal folic acid diet

**Chromosome**	**Start**	**End**	**Gene**	**Total CpG LMFA**	**Total CpG HMFA**	**Methylation difference**	***P value***
**Male**							
Chr1	182625573	182625574	*Mixl1*	28	10	-0.76	0.00
Chr2	25376181	25376182	*Traf2*	24	14	-0.77	0.00
Chr3	37380649	37380650	*Spata5*	14	18	-0.78	0.00
Chr5	145038432	145038433	*Baiap2l1*	58	20	-0.87	0.00
Chr5	145038431	145038432	*Baiap2l1*	28	20	-0.73	0.00
Chr6	63336567	63336568	*Grid2*	12	11	-0.83	0.00
Chr8	119917212	119917213	*Cmip*	12	10	-0.80	0.00
Chr9	49203682	49203683	*Drd2*	14	7	-0.93	0.00
Chr10	86298137	86298138	*Nt5dc3*	20	6	-0.83	0.00
Chr11	89264853	89264854	*4932411E22Rik*	12	8	-1.00	0.00
Chr11	88387073	88387074	*Msi2*	11	10	-0.90	0.00
Chr12	73229241	73229242	*Ccdc175*	19	6	-0.83	0.00
Chr13	117804995	117804996	*Parp8*	12	6	-1.00	0.00
Chr16	49910445	49910446	*Cd47*	8	10	-1.00	0.00
Chr16	96296259	96296260	*Brwd1*	24	8	-0.83	0.00
Chr17	80761785	80761786	*Arhgef33*	20	13	-0.77	0.00
Chr17	64485735	64485736	*Fert2*	9	14	-0.82	0.00
Chr18	36751828	36751829	*Ankhd1*	20	10	-0.90	0.00
Chr19	5690281	5690282	*Map3k11*	20	13	-0.80	0.00
ChrX	98171409	98171410	*Tex11*	10	6	-1.00	0.00
**Female**							
Chr1^a^	106890569	106890570	*Cdh20*	15	14	-0.73	0.00
Chr3	53010469	53010470	*Lhfp*	12	10	-0.92	0.00
Chr4	137849617	137849618	*Kif17*	9	14	-0.86	0.00
Chr4	142704605	142704606	*Prdm2*	15	11	-0.73	0.00
Chr4	21913536	21913537	*6230409E13Rik*	23	10	-0.71	0.00
Chr4	46546442	46546443	*Trim14*	16	11	-0.75	0.00
Chr4	117003265	117003266	*Rnf220*	16	6	-0.83	0.00
Chr5	148775977	148775978	*Mtus2*	20	14	-0.76	0.00
Chr5	37274842	37274843	*Ppp2r2c*	14	17	-0.75	0.00
Chr8	121359642	121359643	*Cdh13*	17	10	-0.74	0.00
Chr9	21909321	21909322	*Cnn1*	10	6	-1.00	0.00
Chr9	44605440	44605441	*Tmem25*	16	10	-0.75	0.00
Chr11	96309521	96309522	*Gm11529*	25	10	-0.88	0.00
Chr11	118204319	118204320	*Timp2*	20	7	-0.81	0.00
Chr11	98634354	98634355	*Nr1d1*	7	15	-0.87	0.00
Chr15	99627500	99627501	*Lima1*	25	22	-0.74	0.00
Chr15	59208541	59208542	*Nsmce2*	9	6	-1.00	0.00
Chr16	49910445	49910446	*Cd47*	10	12	-0.83	0.00
Chr17	26016873	26016874	*Wfikkn1*	36	20	-0.73	0.00
Chr17	28437426	28437427	*Ppard*	8	22	-0.83	0.00

^a^Methylation exclusively in the CpG island. HMFA, high maternal folic acid; LMFA, low maternal folic acid.

### Maternal folic acid alters DNA methylation status in the promoters at CpG Islands

**Figure 2:**
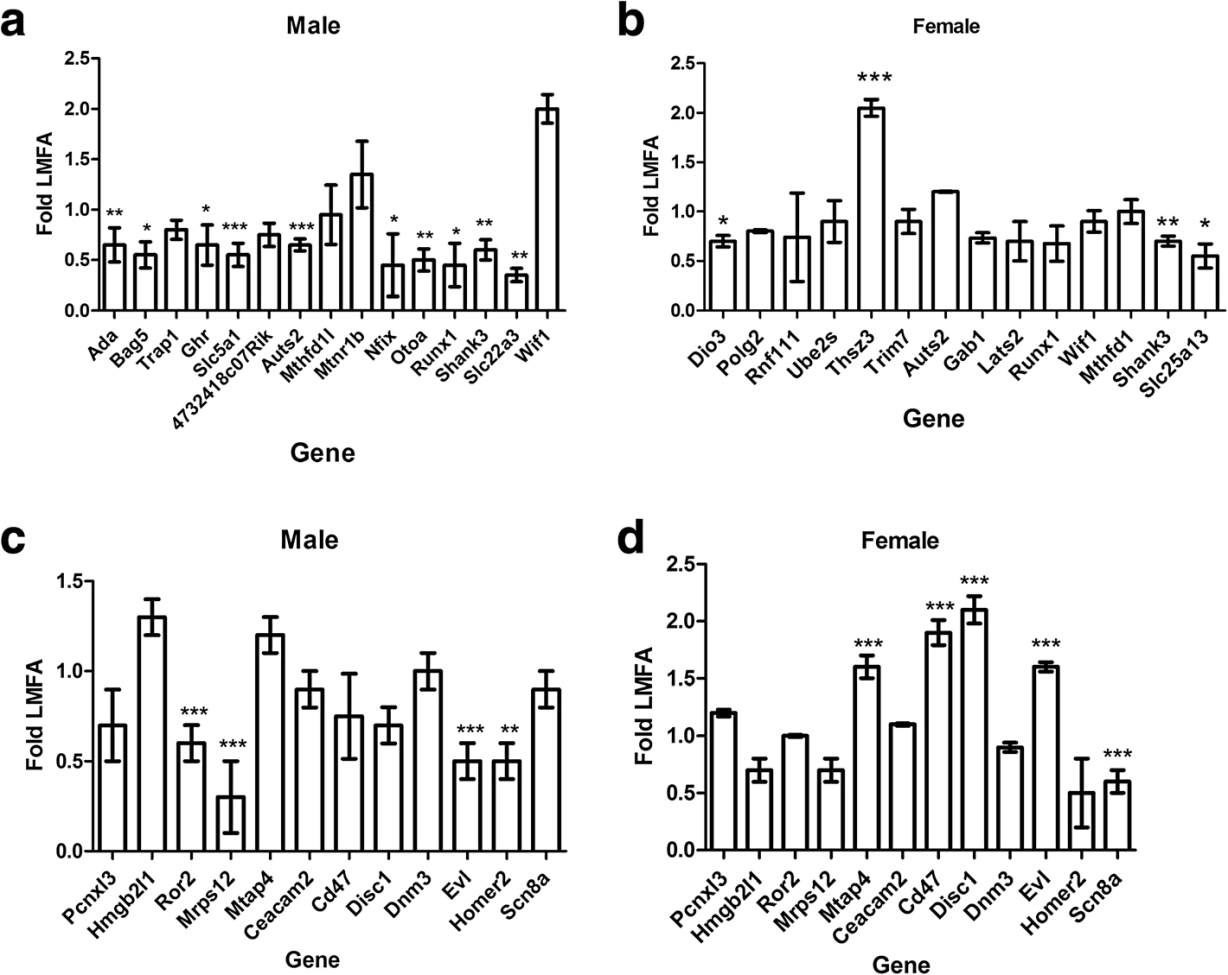
**Relative expression of the genes that exhibited hypermethylation (a, b) and hypomethylation (c, d).** The results were normalized to *Hprt* transcript expression and were expressed as relative values in comparison with corresponding transcripts from low maternal folic acid (LMFA). Results represent mean ± standard deviation (SD); asterisks denote statistically significant change (*P <0.05, **P <0.01, ***P <0.001).

In this study, we found that HMFA throughout gestation resulted in hypermethylation (*P* <0.01) at CpG sites of the promoter region of several genes, including *Ada*, *Bag5*, and *Trap1* in male offspring (Table 1), leading to downregulation of the expression of *Ada* and *Bag5* and no such alterations in expression level of *Trap1* (Figure 2a). In female pups, HMFA also resulted in hypermethylation at CpG sites in the promoter region of the genes *Dio3*, *Polg2*, *Rnf111*, and *Ube2s*, including several other genes (Table 1). Quantitative real time reverse transcription-polymerase chain reaction (qRT-PCR) analysis revealed that the expression of *Dio3* was significantly downregulated and that, in contrast, the expression of *Polg2*, *Rnf111*, and *Ube2s* remained unchanged in female pups from HMFA compared with that of LMFA (Figure 2b). To further reveal the impact of maternal FA, we assessed whether HMFA resulting in hypomethylation in the promoter regions of CpG islands altered the expression levels of those genes as well. We tested the expression of several genes in male (*Pcnxl3*, *Hmgb21l*, and *Ror2*) and female (*Mrps12*, *Ceacam2*, and *Mtap4*) pups (Table 1, Figure 2c, d). In male pups from HMFA, the expressions of *Ror2* and *Mrps12* were significantly downregulated, and in female pups, the expression of *Mtap4* was significantly upregulated in comparison with LMFA. Interestingly, although the methylation level of *Mrps12* did not show any significant change in male pups from HMFA, the expression was significantly downregulated. In contrast, the expression of *Mrps12* in female pups from HMFA showed no difference in expression level, although significant methylation changes were observed. However, the expression analysis of several other genes—*Pcnxl3*, *Hmgb21l*, *Mtap4*, and *Ceacam2*—has shown no significant changes in both the genders from HMFA. The results of our findings suggest that maternal FA modulates the methylation pattern of the offspring genome, and considering the role of maternal nutrition in early neural development, such changes in methylation patterns in promoter CpG sites due to HMFA may have long-term influences on neuronal organization and ultimately on behavioral phenotypes.

### Maternal folic acid alters DNA methylation status in the promoters at non-CpG sites

To extend our findings, we then analyzed whether gestational FA modulates the methylation pattern of non-CpG sites. In this study, we obtained 89% coverage in non-CpG sites (both CHH and CHG context, where H = A, C, or T). The overall distribution of methylation level in the non-CpG sites is shown in Additional file 8: Figures S10 and 11. We identified approximately 1,000 differentially methylated (both hyper- and hypo-methylation) sites (*P* <0.05) in both CHH and CHG contexts in the offspring genome from the HMFA group (Additional file 2: Table S2, Additional file 3: Table S3, Additional file 9: Table S4, Additional file 4: Table S5, and Additional file 5: Table S6). For example, zinc finger proteins *Zfp608* and ephrin receptor *Epha6* in male offspring and *Zfp719*, *Zfp804b*, *Zfp128* and calcium channel *Cacna1g* in female offspring are a few of the many genes that were hypermethylated (*P* <0.05) in the non-CpG promoter sites (Additional file 9: Table S4). Furthermore, we tested expression levels of several genes (*Ghr*, *Slc5a1*, and *4732418C07Rik*) in male pups and (*Tshz3* and *Trim7*) in female pups with Quantitative real time reverse transcription-polymerase chain reaction (qRT-PCR). The results showed that the expression of EF-hand calcium-binding domain 14 (*4732418c07Rik*) remain unchanged; in contrast, the expression of sodium-dependent glucose transporter (*Slc5a1*), which exhibited hypermethylation in CHG sites, and growth hormone receptor (*Ghr*) [14], which exhibited hypermethylation at both CHG and CHH sites in the promoter region, were downregulated in male offspring from HMFA (Figure 2a, Additional file 9: Table S4). A representative figure depicting the methylation status of a non-CpG (CHG) hypermethylation at *Slc5a1* promoter of male offspring from the data uploaded in the UCSC Genome Browser is shown in Additional file 10: Figure S12.

### Maternal folic acid alters DNA methylation pattern in the gene body

**Figure 3:**
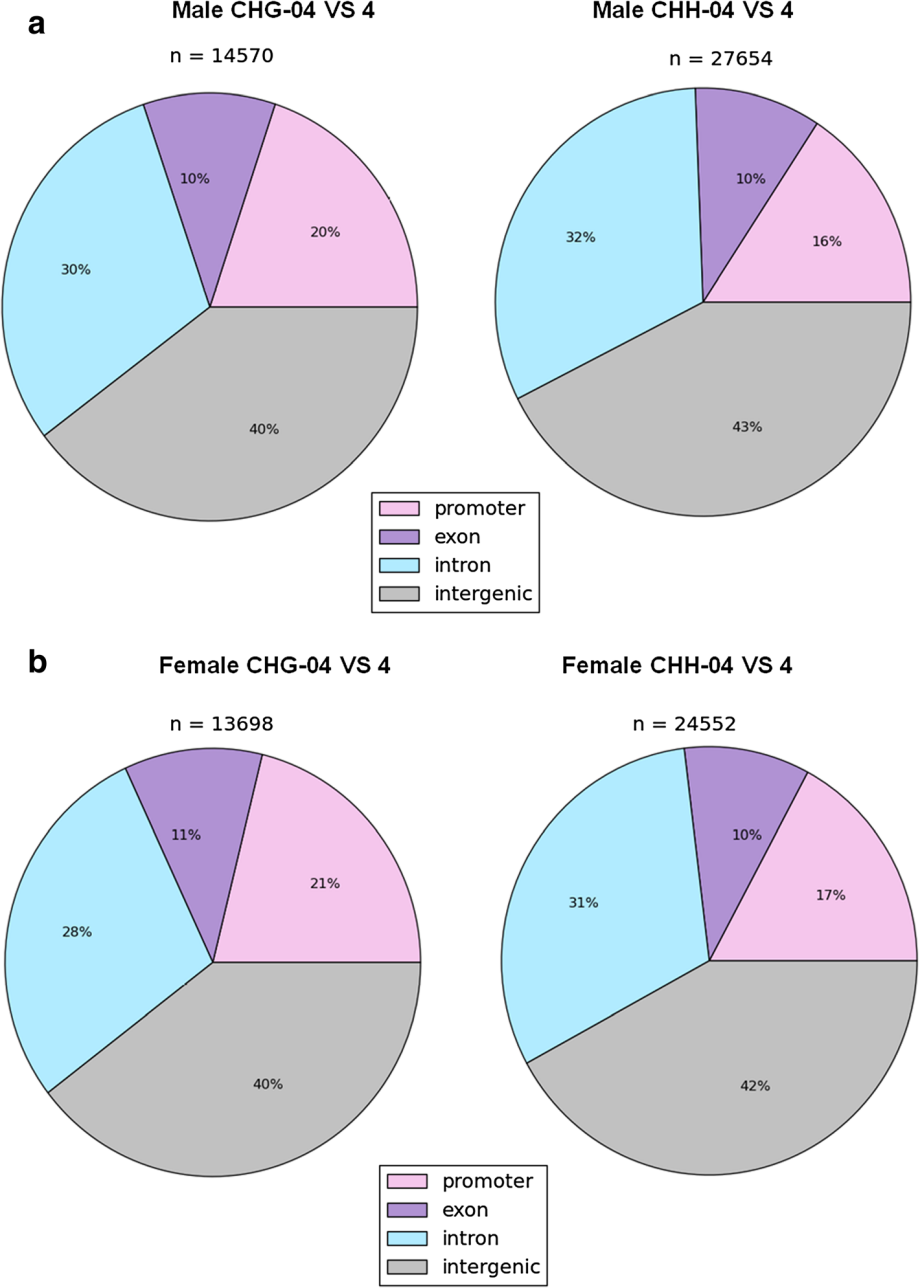
**Distribution of differentially methylated sites in non CpG (CHG/CHH) sites. (a)** Male low maternal folic acid (LMFA) versus high maternal folic acid (HMFA). **(b)** Female LMFA versus HMFA.

An interesting aspect of our data is the pattern of methylation in both CpG and non-CpG sites in gene bodies. The majority of the non-CpG associated DMRs were either intergenic or in introns, whereas 10% to 11% were in exons, and approximately 16% to 21% were in promoter regions in both male and female pups from HMFA (Figure 3a, b). The overall distribution of methylation level in exons is shown in Additional file 11: Figure S13 and S14. Several candidate autism susceptibility genes [15] were hypermethylated (*P* <0.05) in the HMFA, including *Shank3*, *Cacana1g*, *Gtf2i*, *Rapgef4*, and *Nbea* in male offspring and *Ext1*, *Ube3a*, *Erbb4*, *Grip1*, *Grm8*, *Reeln*, *Shank3*, and *Rbfox1* in female offspring (Additional file 2: Table S2 and Additional file 3: Table S3). In contrast, several candidate autism susceptibility genes were also hypomethylated; for example, *Disc1*, which is known to play a pivotal role in cortical development, and *Scn8a*, which modulates membrane depolarization, were hypomethylated in the gene body in both male and female offspring (*P* <0.05) in the HMFA group (Additional file 4: Table S5 and Additional file 5: Table S6). It is interesting to note that autism susceptibility candidate 2 (*Auts2*) gene exhibited both hyper- and hypo-methylation in the gene bodies of male and female pups from HMFA. Further analysis of methylation profile also revealed hypermethylation in imprinted genes in male (*Slc22a3* and *Ano1*) and in female (*Gab1*, *Calcr*, *Dio3*, and *Slc38a4*) offspring (*P* <0.05) (Additional file 2: Table S2 and Additional file 3: Table S3). On the other hand, imprinted genes *Peg12* and *Slc22a18* in female offspring and cadherin-associated protein *Ctnna3* in both male and female offspring from the HMFA group were hypomethylated (Additional file 4: Table S5 and Additional file 5: Table S6). To verify changes in the expression levels, we tested the mRNA expression of several genes by qRT-PCR. Genes in male pups (*Auts2*, *Mthfd1l*, *Mtnr1b*, *Nfix*, *Otoa*, *Runx1*, *Shank3*, *Slc22a3*, and *Wif1*) and in female pups (*Auts2*, *Gab1*, *Lats2*, *Runx1*, *Wif1*, *Mthfd1*, *Shank3*, and *Slc25a13*) were analyzed. The results revealed that the expressions of *Auts2*, *Nfix*, *Otoa*, *Runx1*, *Shank3*, and *Slc22a3* were significantly downregulated; in contrast, the expression of *Mthfd1l*, *Mtnr1b*, and *Wif1* did not exhibit significant changes as a result of HMFA in comparison with LMFA in male pups (Figure 2a). In female pups from the HMFA group, the expression of *Auts2*, *Gab1*, *Lats2*, *Runx1*, *Wif1*, and *Mthfd1* did not exhibit significant changes; in contrast, the expressions of *Shank3* and *Slc25a13* were significantly downregulated (Figure 2b). We further analyzed the expression of several genes which exhibited hypomethyaltion in the gene body of several genes (*Cd47*, *Disc1*, *Dnm3*, *Evl*, *Sn8a*, and *Homer2*) in both male and female pups from HMFA in comparison with LMFA (Figure 2c, d). The result of gene expression in male pups revealed significant downregulation in the expression of *Evl* and *Homer2* whereas no such significant differences in expression of *Cd47*, *Disc1*, *Dnm3*, and *Sn8a* were observed. In contrast, in female pups, the expression of *Sn8a* was downregulated and the expressions of *Cd47*, *Disc1*, and *Evl* were upregulated whereas the expression of *Dnm3* did not exhibit any change in expression.

### Maternal folic acid modulates sex-specific alterations in global DNA methylation in the offspring’s cerebral hemispheres

We further investigated the impact of maternal FA during gestation on epigenetic alterations throughout the genome in a sex-specific manner. Comparison between male and female pups’ cerebral hemispheres from mothers fed an LMFA or HMFA revealed significant sexual dimorphism for global DNA methylation. Approximately 21% of the CpG sites were differentially methylated between males and females from both LMFA (n = 55,640) and HMFA (n = 45,634). The distributions of CpG-island and non-CpG island associated methylations between male and female are shown in Additional file 12: Figure S15 and Additional file 13: Figure S16a,b. The majority of the DMRs in CpG or non-CpG island between males and females from LMFA and HMFA were in intergenic or in introns, whereas 9% to 20% in exons and 10% to 21% were in promoter regions. Further analysis of the data revealed striking sexual dimorphism in methylation patterns of numerous genes as a result of both LMFA and HMFA (Additional file 14: Table S7, Additional file 15: Table S8, Additional file 16: Table S9, and Additional file 17: Table S10). The correlation of the distribution of methylation ratios between male and female pups for the corresponding sites in LMFA and HMFA is shown in Additional file 18: Figure S17a,b,c and Additional file 19: Figure S18a,b,c, and the hexbin plot (Additional file 20: Figure S19a,b,c) shows the distribution of the overlapped sites between genders of LMFA and HMFA from total significant (*P* <0.05) differential methylation sites. To evaluate whether the expressions of the tested genes in this study were biased by gender, we analyzed the expression of several genes between male and female pups from LMFA and HMFA, which exhibited changes in methylation profile. First we compared the expression of genes *Trap1*, *Runx1*, *Scn8a*, and *Cd47* (hypermethylated) and *Auts2* and *Rnf111* (hypomethylated) in female pups from LMFA in comparison with LMFA from male pups (Additional file 21: Figure S20a). The results show that the expressions of *Trap1* and *Cd47* were significantly downregulated and the expression of *Runx1* was upregulated, whereas the expressions of *Scn8a*, *Auts2*, and *Rnf111* remained unchanged. Similarly, we compared the expression of genes *Dio3*, *Trim7*, *Shank3*, *Slc25a13*, *Auts2*, *Disc1*, and *Dnm3* (hypermethylated) and *Bag5*, *Ghr*, *Ror2*, and *Runx1* (hypomethylated) in female pups from HMFA in comparison with HMFA from male pups (Additional file 21: Figure S20b). The results show that the expressions of *Dio3*, *Trim7*, *Shank3*, *Slc25a13*, *Auts2*, *Disc1*, *Ror2*, and *Runx1* were upregulated, whereas the expressions of *Bag5* and *Ghr* were downregulated and the expression of *Dnm3* remained unchanged. These results show that expressions of several genes are biased between male and female pups both in the basal level (LMFA) and as a result of HMFA.

Moreover, to control and maintain the sexual-dimorphism hypothesis, we further analyzed the expression of several tested genes, which exhibited sexual dimorphism in methylation profile. For example, genes which exhibited changes in methylation level in male pups as a results of HMFA are tested in female pups (no changes in methylation profile); similarly, genes which exhibited changes in methylation level in female pups as a result of HMFA are tested in male pups (no change in methylation profile). The expression analysis of genes *Dio3*, *Polg2*, *Rnf111*, *Ube2s*, *Thsz3*, *Trim7*, *Gab1*, *Lats2*, and *Slc25a13* (hypermethylated, in female pups) and *Mrps12*, *Mtap4*, and *Ceacam2* (hypomethylated in female pups) were tested in male pups from HMFA in comparison with male pups from LMFA (Additional file 22: Figure S21a). The expression of *Rnf111* was upregulated and the expression of *Mrps12* (Figure 2c) was downregulated, whereas other tested genes did not exhibit any significant changes in male pups. It is interesting to note that the expressions of *Dio3* and *Slc25a13* were significantly downregulated and the expressions of *Thsz3* and *Mtap4* were significantly upregulated in female pups from HMFA in comparison with LMFA (Figure 2b, d). Similarly, we tested the expression of several genes in female pups from HMFA which exhibited no changes in methylation compared with female pups from LMFA. For example, the expressions of genes *Ada*, *Bag5*, *Trap1*, *Ghr*, *4732418C07Rik*, *Mthfd1l*, *Nfix*, *Otoa*, and *Slc22a3* (hypermethylated in male pups) and *Pcnxl3*, *Hmgb2l1*, and *Ror2* (hypomethyalted, in male pups) were tested. The results showed (Additional file 22: Figure S21b) that the expression of *Ada*, *Bag5*, and *Slc22a3* were unchanged in female pups from HMFA in comparison with LMFA. In contrast, the expressions of *Ghr* and *Nfix* were upregulated in female pups from HMFA. It is interesting to note that the expression of *Otoa* is downregulated whereas the expressions of *Trap1*, *4732418C07Rik*, *Mthfd1l, Pcnxl3*, *Hmgb2l1*, and *Ror2* remained unchanged in both male and female pups as results of HMFA. These results show that the expressions of several tested genes are sexually biased as a result of HMFA. Additionally, we have evaluated that the methylation patterns were affected in cis-alteration in CpG and CHG contexts in both males and females (Additional file 23: Table S11).

## Discussion

In a fertilized egg, global DNA demethylation followed by remethylation occurs to reprogram the maternal and paternal genomes for efficient regulation of gene expression. Certain genes are turned on and off at particular time intervals, and any disruption of such highly orchestrated methylation regulation may impact gene expression. The fetal epigenome is most vulnerable during this period of development to epigenetic modifiers in the maternal microenvironment. Because lifestyle and the level of nutrition available during gestation play an important role in the offsprings’ gene regulation, maternal FA consumed could dictate the establishment of epigenetic patterns of the offspring. In this study, we found that HMFA during gestation resulted in substantial changes in the methylation profile of the offspring’s cerebral hemispheres. Over the years, numerous studies have implicated several candidate autism susceptibility genes with a logical focus on the affected child. However, a consistent picture of specific susceptibility loci has thus far met with limited success [16-18]. In humans, FA during gestation has been shown to prevent autism or NTDs [19-21]. Intriguingly, our results showed hypermethylation but no hypomethylation in the promoter region of candidate autistic and imprinted genes in the offspring from the HMFA group. In this study, we found that HMFA resulted in hypermethylation (*P* <0.01) at the CpG sites of the promoter region of *Ada* in male offspring. It is noteworthy that previous evidence had suggested decreased *Ada* activity in autistic subjects [22-24] and in a severe combined immune deficiency syndrome [25,26]. Thus, such changes in methylation patterns in promoter CpG sites due to HMFA may have long-term influences on neuronal organization and behavioral phenotypes. In addition, epigenetic modifications of the imprinted genes such as *Dio3* can result in clinically significant phenotypes. It will be of interest to examine the impact of the dose and duration of maternal FA and the consequences of these epigenetic effects. Similar to hypermethylation, we observe hypomethylation in promoter regions of CpG sites in both male and female pups from HMFA in comparison with LMFA. The expression of *Ror2* (hypomethylated in male pups) which plays a role during neurogenesis of the developing neocortex [27] was significantly downregulated in male pups, but no such changes were observed in female pups. Moreover, the expression of *Mtap4* (hypomethylated in female pups) that has been shown to play a role in the central nervous system and regulation of the microtubule-dependent transport [28] was upregulated significantly in female pups from HMFA in comparison with LMFA, but the expression in male pups did not exhibit such significant changes. Intriguingly, the expression of *Mrsps12* that exhibited hypomethylation in female pups from HMFA remained unchanged in comparison with LMFA. In contrast, the expression of *Mrps12* in male pups was significantly downregulated in HMFA in comparison with LMFA, although no such changes in methylation pattern were observed. Of note, *Mrps12* is a major component of the ribosomal accuracy center and has been shown to play a role in sensorineural deafness [29].

Recently, the methylation of non-CpG regions and its role in transcriptional repression have received greater attention [30,31], and single-base resolution maps of the human genome have revealed a substantial presence of methylated cytosine residues in non-CpG contexts [32]. In this study, we found substantial differential methylation in non-CpG regions in gene promoters from newborns of HMFA and confirmed the variations in the expression of several genes. Notably, studies using the brain tissue from an Alzheimer disease model [33], fetal brain [34,35], adult tissues [36,37], and early embryo [38] have shown that methylation in both CpG islands and non-CpG regions correlates with the expression of several genes. Thus, variation of methylation in the non-CpG regions as a result of HMFA may modulate epigenetic-mediated transcriptional repression, although a direct causal connection cannot be established with our data. Further analysis of the methylation profile has shown substantial differential methylation in the gene body of the offspring DNA. This finding builds on growing evidence that maternal adversity during gestation induces unbiased epigenetic changes in offspring genome. Although aberrant methylation in gene promoter regions is known to be linked with altered gene expression, the effect of hypermethylation in the gene body is unclear and inconclusive [39]. Significant evidence indicates that gene body methylation is a general feature of highly expressed genes in human cell lines [40-42]. In contrast, a recent study in a mouse model has revealed that differential gene body methylation generally resulted in downregulation of gene expression [43]. We found both up- and downregulation in the expression of transcripts that exhibited hypermethylation in the gene body of the offspring from HMFA. Our study suggests that the relationship between gene body methylation and transcriptional level may be more complicated than previously thought and, perhaps, underappreciated. Intriguingly the mRNA expression of *Shank3* was significantly downregulated in both male and female pups; in contrast, the expression of *Auts2* is downregulated in male pups but not in female pups. The genes *Auts2* and *Shank3* are associated with autism spectrum disorders and other neurological diseases [44,45], and in this study both of the genes exhibited an alteration in methylation pattern in the gene body.

Our findings showed several distinct DMRs to be acting in a sexually dimorphic manner, similar to a recent study on imprinted genes in the placenta [46]. The relevance of epigenetic mechanisms in developing several complex diseases is sex-biased, and numerous studies have shown that during the developing windows of life the environmental factors, including nutrition during prenatal and postnatal life, influence epigenetic modulation in a sex-related manner [47-50]. Several studies in humans have further shown that various late-onset diseases are sex-biased and are highly related to maternal diet and body condition during pregnancy [51-53]. In this study, in the mouse model, we found that the expressions of several genes as a result of HMFA are highly biased in expression depending upon the gender. Furthermore, analysis of the methylation profile and gene expression between LMFA of male and female pups and between HMFA of male and female pups reveals striking sexual dimorphism. One possibility of such sexual dimorphism is the alterations in the uterine environment because of changes in FA level, and the methylation of imprinted genes may fine-tune selective events specific to one sex during developmental programming. Thus, the results of our study further highlight the relevance of studying both sexes in experimental models of maternal diet and may provide critical insight regarding the influence of FA in programming sex-biased methylation pattern.

It is paradoxical that the methylation profile of our findings shows a substantial hypomethylation to be present in the offspring DNA, even after supplementing HMFA. This finding indicates that the amount of gestational extracellular FA or cofactor required for the synthesis of S-adenosylmethionine probably can induce the site- and gene-specific nature of the methylation level in the offspring DNA, and probably the DNA methylation status is also dependent upon methylenetetrahydrofolate reductase (MTHFR) activity and not only on the folate status alone [54,55]. Moreover, because DNA methylation is a distinguishing feature that varies between cell types, specifically various neuronal populations, the variation in methylation (hypo/hyper) profile of our sample may be due to the cellular heterogeneity of the cerebral hemisphere [56-58].

## Methods

### Mice strain and feeding

Mice in this study were handled according to the protocol reviewed and approved by the Institute for Basic Research Institutional Animal Care and Use Committee. Adult, 8- to 10-week-old C57BL/6 J mice were used in all the experiments. Throughout the experimental procedure, controlled temperature and a fixed lighting schedule were maintained in the room. Given that most current commercial mice chow already contains quite high amounts of FA (2–3 mg/kg diet) and thus is unsuitable for the current studies, we developed a custom diet for this study. One week prior to mating, female mice were fed with a custom AIN-93G amino acid-based diet (Research Diet, Inc.), having FA at 0.4 mg/kg (n = 8–12), while the test group received FA at 4 mg/kg (n = 8–12). The diet was continued throughout the entire period of gestation.

### Tissue collection and DNA extraction

At postnatal day 1, six pups (n = 6, segregated by gender), all from different dams, in each diet group were sacrificed. Cerebral hemisphere tissues were pooled (n = 3/gender) for the 0.4 mg group: three male pups (each from an independent dam) and three female pups (each from an independent dam), for a total of six pups (n = 6). Tissues from six pups (n = 6, segregated by gender) from the 4 mg group were similarly processed. DNA was extracted from pooled cerebral hemispheres (n = 3/gender per group) with the Epicentre MasterPure DNA purification kit (Epicentre Biotechnologies, Madison, WI, USA) in accordance with the protocol of the manufacturer. After re-suspension in TE buffer, the DNA concentration was measured by using NanoDrop ND-1000 (Thermo Scientific, Wilmington, DE, USA).

### Library construction

To perform a genome-wide DNA methylation analysis, libraries were prepared from 200 to 500 ng of genomic DNA digested sequentially with 60 units of TaqI and 30 units of MspI (New England Biolabs, Ipswich, MA, USA). The resulting size-selected TaqI-MspI fragments (40–120 bp and 120–350 bp) were filled in, and 3′-terminal-A extended, extracted with DNA Clean & Concentrator™ kit (Zymo Research, Irvine, CA, USA). Ligation of selected fragments to pre-annealed adapters containing 5′-methyl-cytosine instead of cytosine was performed by using the Illumina DNA preparation kit in accordance with the protocol of the manufacturer (Illumina Inc., San Diego, CA, USA). Purified, adaptor-ligated fragments were then bisulphite-treated by using the EZ DNA Methylation-Direct™ Kit (Zymo Research). Preparative-scale PCR was performed with the resulting fragments followed by purification of PCR products with DNA Clean & Concentrator™ (Zymo Research). Final size selection of the purified PCR products was performed by using 4% NuSieve 3:1 agarose gel. SYBR-green-stained gel slices of adapter-ligated fragments (130–210 bp or 210–460 bp in size) were excised, and library material was recovered by using the Zymoclean™ Gel DNA Recovery Kit (Zymo Research). Sequencing was performed on an Illumina HiSeq genome analyzer.

### Sequence alignments and data analysis

Using standard base-calling software, sequence reads from bisulfite-treated EpiQuest libraries were identified. Further analysis was performed by using a Zymo Research proprietary analysis pipeline. First, residual cytosines (Cs) in each read were converted to thymines (Ts), with each conversion noted for subsequent analysis. From the 50-bp ends of each computationally predicted MspI-TaqI fragment (40- to 350-bp size range), a reference sequence database was constructed. All Cs in each fragment end were then converted to Ts; only the C-poor strands are sequenced in the RRBS (reduced representation bisulfite sequencing) process. Then, using Bowtie software (http://bowtie-bio.sourceforge.net/index.shtml), the converted reads were aligned to the converted reference. The number of mismatches in the induced alignment was then counted between the unconverted read and reference, ignoring cases in which a T in the unconverted read was matched to a C in the unconverted reference. For a given read, the best alignment was kept if the second-best alignment had two more mismatches; otherwise, the read was discarded as non-unique. The methylation level of each sampled C was estimated as the number of reads reporting a C, divided by the total number of reads reporting a C or T. For each CpG site, Fisher’s exact test or *t* test was performed, which covered at least five reads. Also, promoter, gene body, and CpG island annotations were added for each CpG. The software pipeline is implemented in Python. All the procedures above were carried out in the Zymo Epigentic Core Services (Zymo Research).

### RNA preparation and quantitative real time reverse transcription-polymerase chain reaction analysis

At postnatal day 1, six pups (n = 6, segregated by gender) from different dams in each diet group were sacrificed. Cerebral hemisphere tissues were pooled (n = 3/gender) for the 0.4 mg group: three male pups (each from an independent dam) and three female pups (each from an independent dam), for a total of six pups (n = 6). Tissues from six pups (n = 6) from the 4 mg group were similarly processed. Considering the degree of inter-variability, RNA extractions were repeated from a different batch (pooled samples, n = 3 for each group/gender, with each mouse from a different dam). Total RNA was extracted by lysing the cells with Trizol reagent (Invitrogen Life Technologies, Inc., Carlsbad, CA, USA). Further purification of RNA was carried out using RNeasy kit (Qiagen, Valencia, CA, USA) in accordance with the instructions of the manufacturer. On-column DNase digestion for each sample was performed to remove any DNA contamination. The quality of RNA was assessed by measuring the absorbance ratio at 260/280 nm by using NanoDrop ND-1000 (Thermo Scientific). The integrity of RNA was further assessed by formaldehyde-gel electrophoresis. Quantitative real time reverse transcription-polymerase chain reaction amplifications were performed with either One-Step iScript kit (Bio-Rad, Hercules, CA, USA) or Two-step kit in which the first-strand cDNA from each sample was synthesized from 1 μg total RNA by using the First-Strand cDNA Synthesis kit (Affymetrix, Santa Clara, CA, USA) in accordance with the protocol of the manufacturer. qRT-PCR was performed by using the Mastercycler ep Realplex system (Eppendorf AG, Hamburg, Germany) in combination with the RT^2^ SYBRGreen PCR Master Mix (Qiagen). Each reaction was run in duplicate and repeated at least two times each from different batches of RNA (pooled n = 3 per batch/gender). *Hprt1* was used as endogenous control for amplification. Relative gene expression was calculated by using the Pfaffl method [59]. Primers used for qRT-PCR are listed in Additional file 24: Table S12. Statistical analysis was done by using Prism Software (GraphPad, San Diego, CA, USA). Values are presented as means ± standard deviation, and numerical results are presented considering *P* <0.05 as significant.

## Conclusions

In summary, we have identified substantial DMRs in the cerebral hemispheres of the offspring, revealing that the HMFA diet causes epigenetic modifications. A key finding of this study is the presence of DMRs in non-CpG regions, along with CpG sites at single-base resolution, as a result of HMFA. Because numerous studies have shown that abnormalities in the frontal lobes impact brain development and autism [60,61], our study’s findings could provide a completely novel insight into the etiology of complex developmental disorders and foster the development of corrective strategies. However, we do not rule out the limitation of our study that the methylation and gene expression do not necessarily indicate change in function, and thus further studies are required in a larger number of samples to verify the functional outcome and phenotypes.

## Abbreviations

C: cytosine; DMR: differentially methylated region; FA: folic acid; HMFA: high maternal folic acid; LMFA: low maternal folic acid; NTD: neural tube defect; qRT-PCR: quantitative real time reverse transcription-polymerase chain reaction; T: thymine.

## Competing interests

The authors declare that they have no competing interests.

## Authors’ contributions

SB helped to perform the experiments, to analyze the data, and to write the paper. SK and KKC helped to perform the experiments. MAJ helped to perform the experiments, to analyze the data, to write the paper, and to conceptualize the research. MJF helped to analyze the data. WTB helped to analyze the data and to conceptualize the research. All authors read and approved the final manuscript.

## Supplementary Material

Additional file 1: Table S1 Descriptive statistics of the mapping of methylation profile of the cerebral hemispheres of offspring from low maternal folic acid (LMFA) and high maternal folic acid (HMFA) diets. Click here for file

Additional file 2: Table S2 Genes in male offspring from high maternal folic acid (HMFA) diet, which were enriched with methylation in the gene body and other chromosomal regions in CpG/CHG/CHH contexts. Click here for file

Additional file 3: Table S3 Genes in female offspring from high maternal folic acid (HMFA), which were enriched with methylation in the gene body and other chromosomal region in CpG/CHG/CHH contexts. Click here for file

Additional file 4: Table S5 Genes that were hypomethylated in the CpG/CHG/CHH contexts of the male offspring from high maternal folic acid (HMFA) diet. Click here for file

Additional file 5: Table S6 Genes that were hypomethylated in the CpG/CHG/CHH region of the female offspring from high maternal folic acid (HMFA) diet. Click here for file

Additional file 6: Figures S1-S6 Scatter plot representing the distribution of the methylation ratio for corresponding sites of low maternal folic acid (LMFA) versus high maternal folic acid (HMFA). Pearson’s correlation coefficient is denoted in the center of each scatter plot. Click here for file

Additional file 7: Figures S7-S9 Hexbin plot representing the overlapped sites in CpG (n = 4,378), CHG (n = 71), and CHH (n = 149) regions between male and female pups from high maternal folic acid (HMFA) in comparison with low maternal folic acid (LMFA) from total significant (*P* <0.05) differential methylation sites. Each dot in hexbin plot is one of the overlapped sites. The colors blue, green, yellow and red represent the dot density from lower to higher order in accordance with the prevalence of the overlapping sites. Click here for file

Additional file 8: Figures S10, S11 Box plot illustrating the methylation levels across non-CpG islands of the cerebral hemispheres from male pups (S10) having low maternal folic acid (LMFA) (n = 36,319 and median = 0.65) and high maternal folic acid (HMFA) (n = 36,319 and median = 0.73) and from female pups (S11) having LMFA (n = 48,438 and median = 0.66) and HMFA (n = 48,438 and median = 0.69) as assessed by reduced representation bisulfite sequencing (RRBS). Boxes are 25th and 75th quartiles; horizontal yellow bar in the middle represents the median DNA methylation value. Whisker indicates the 5th and 95th percentiles. Click here for file

Additional file 9: Table S4 Genes in male and female offspring from high maternal folic acid (HMFA), which were enriched with methylation in the gene promoters in CHG/CHH. Click here for file

Additional file 10: Figure S12 A representative figure of the data uploaded in the University of California at Santa Cruz (UCSC) Genome Browser. Comparison of DNA methylation patterns in the offspring’s cerebral hemisphere from low maternal folic acid (LMFA) and high maternal folic acid (HMFA). Example of the *Slc5a1* gene in chromosome 5 that was differentially methylated in the CHG contexts of promoter region of male offspring. Yellow color bars indicate gain of methylation. Click here for file

Additional file 11: Figures S13, S14 Box plot illustrating the methylation levels across exons of the cerebral hemispheres from male pups (S13) having low maternal folic acid (LMFA) (n = 8,136 and median = 0.42) and high maternal folic acid (HMFA) (n = 8,136 and median = 0.5) and from female pups (S14) having LMFA (n = 10,335 and median = 0.44) and HMFA (n = 10,335 and median = 0.5) as assessed by reduced representation bisulfite sequencing (RRBS). Boxes are 25th and 75th quartiles; horizontal yellow bar in the middle represents the median DNA methylation value. Whisker indicates the 5th and 95th percentiles. Click here for file

Additional file 12: Figure S15 Distribution of differentially methylated sites in CpG island sequences between males and females from (a) low maternal folic acid (LMFA) and (b) high maternal folic acid (HMFA). Click here for file

Additional file 13: Figure S16 Distribution of differentially methylated sites in non CpG island sequences between males and females from (a) low maternal folic acid (LMFA) and (b) high maternal folic acid (HMFA). Click here for file

Additional file 14: Table S7 Genes in female offspring from low maternal folic acid (LMFA) diet, which were enriched with methylation, compared with male offspring in the CpG/ CHG/CHH contexts. Click here for file

Additional file 15: Table S8 Genes in female offspring from low maternal folic acid (LMFA) diet, which were hypomethylated, compared with male offspring in the CpG/CHG/CHH contexts. Click here for file

Additional file 16: Table S9 Genes in female offspring from high maternal folic acid (HMFA) diet, which were enriched with methylation, compared with male offspring in the CpG/CHG/CHH contexts. Click here for file

Additional file 17: Table S10 Genes in female offspring from high maternal folic acid (HMFA) diet, which were hypomethylated, compared with male offspring in the promoter region in CpG/CHG/CHH contexts. Click here for file

Additional file 18: Figures S17 (a, b, c) Scatter plot representing the distribution of the methylation ratio for corresponding sites of low maternal folic acid (LMFA) male versus LMFA female in CpG/CHG/CHH regions. Pearson’s correlation coefficient is denoted in the center of each scatter plot. Click here for file

Additional file 19: Figures S18 (a, b, c) Scatter plot representing the distribution of the methylation ratio for corresponding sites of high maternal folic acid (HMFA) male versus HMFA female in CpG/CHG/CHH regions. Pearson’s correlation coefficient is denoted in the center of each scatter plot. Click here for file

Additional file 20: Figures S19 (a, b, c) Hexbin plot representing the overlapped sites in CpG (n = 6,085), CHG (n = 96), and CHH (n = 154) regions between male and female pups from LMFA compared with high maternal folic acid (HMFA) pups from total significant (*P* <0.05) differential methylation sites. Each dot in hexbin plot is one of the overlapped sites. The colors blue, green, yellow, and red represent the dot density from lower to higher order in accordance to the prevalence of the overlapping sites. Click here for file

Additional file 21: Figure S20a Quantitative real time reverse transcription-polymerase chain reaction (qRT-PCR ) showing relative expression of the transcripts of genes in female pups that exhibited hypermethylation or hypomethyaltion in the cerebral hemispheres in comparison with male pups from low maternal folic acid (LMFA). The results were normalized to *Hprt* transcript expression and were expressed as relative values in comparison with corresponding transcripts from male LMFA. Results represent mean ± standard deviation (SD); asterisks denote statistically significant change (*P <0.05, **P <0.01, ***P <0.001). **Figure S20b.** qRT-PCR showing relative expression of the transcripts of genes in female pups that exhibited hypermethylation or hypomethyaltion in the cerebral hemispheres in comparison with male pups from high maternal folic acid (HMFA). The results were normalized to *Hprt* transcript expression and were expressed as relative values in comparison with corresponding transcripts from male HMFA. Results represent mean ± SD; asterisks denote statistically significant change (*P <0.05, **P <0.01, ***P <0.001). Click here for file

Additional file 22: Figure S21a Quantitative real time reverse transcription-polymerase chain reaction (qRT-PCR) showing relative expression of the transcripts of genes in male pups from high maternal folic acid (HMFA) that exhibited no alterations in the methylation profile in promoter and gene body in the cerebral hemispheres compared with low maternal folic acid (LMFA). The results were normalized to *Hprt* transcript expression and were expressed as relative values in comparison with corresponding transcripts from LMFA. Results represent mean ± standard deviation (SD); asterisks denote statistically significant change (*P <0.05, **P <0.01, ***P <0.001). **Figure S21b.** qRT-PCR showing relative expression of the transcripts of genes in female pups from HMFA that exhibited no alterations in the methylation profile in promoter and gene body in the cerebral hemispheres compared with LMFA. The results were normalized to *Hprt* transcript expression and were expressed as relative values in comparison with corresponding transcripts from LMFA. Results represent mean ± SD; asterisks denote statistically significant change (*P <0.05, **P <0.01, ***P <0.001). Click here for file

Additional file 23: Table S11 List of top 100 sites, sorted by *P* value, which exhibited cis-alterations in male and female offspring in the CpG/CHG contexts. Click here for file

Additional file 24: Table S12 List of primers used for quantitative real time reverse transcription-polymerase chain reaction (qRT-PCR) in this study. Click here for file

## References

[B1] Bailey LB Gregory JF III Folate metabolism and requirements J Nutr 1999 129 779 782 10203550 10.1093/jn/129.4.779

[B2] Friso S Choi SW Gene-nutrient interactions in one-carbon metabolism Curr Drug Metab 2005 6 37 46 10.2174/1389200052997339 15720206

[B3] Chanarin I Macgibbon BM O’Sullivan WJ Mollin DL Folic-acid deficiency in pregnancy, The pathogenesis of megaloblastic anaemia of pregnancy Lancet 1959 2 634 639 13809100 10.1016/s0140-6736(59)91409-6

[B4] Hibbard BM Hibbard ED Jeffcoate TN Folic acid and reproduction Acta Obstet Gynecol Scand 1965 44 375 400 10.3109/00016346509155874 5863074

[B5] Smithells RW Sheppard S Schorah CJ Vitamin deficiencies and neural tube defects Arch Dis Child 1976 51 944 950 10.1136/adc.51.12.944 1015847 PMC1546171

[B6] U.S. Preventive Services Task Force Folic acid for the prevention of neural tube defects: U.S. Preventive Services Task Force recommendation statement Ann Intern Med 2009 150 626 631 19414842 10.7326/0003-4819-150-9-200905050-00009

[B7] Haberg SE London SJ Stigum H Nafstad P Nystad W Folic acid supplements in pregnancy and early childhood respiratory health Arch Dis Child 2009 94 180 184 10.1136/adc.2008.142448 19052032 PMC3612898

[B8] Whitrow MJ Moore VM Rumbold AR Davies MJ Effect of supplemental folic acid in pregnancy on childhood asthma: a prospective birth cohort study Am J Epidemiol 2009 170 1486 1493 10.1093/aje/kwp315 19880541

[B9] Steegers-Theunissen RP Obermann-Borst SA Kremer D Lindemans J Siebel C Steegers EA Slagboom PE Heijmans BT Periconceptional maternal folic acid use of 400 microg per day is related to increased methylation of the IGF2 gene in the very young child PLoS One 2009 4 e7845 10.1371/journal.pone.0007845 19924280 PMC2773848

[B10] Junaid MA Kuizon S Cardona J Azher T Murakami N Pullarkat RK Brown WT Folic acid supplementation dysregulates gene expression in lymphoblastoid cells-implications in nutrition Biochem Biophys Res Commun 2011 412 688 692 10.1016/j.bbrc.2011.08.027 21867686

[B11] Heid MK Bills ND Hinrichs SH Clifford AJ Folate deficiency alone does not produce neural tube defects in mice J Nutr 1992 122 888 894 1552363 10.1093/jn/122.4.888

[B12] Docherty SJ Davis OS Haworth CM Plomin R Mill J Bisulfite-based epityping on pooled genomic DNA provides an accurate estimate of average group DNA methylation Epigenetics Chromatin 2009 2 3 10.1186/1756-8935-2-3 19284538 PMC2657899

[B13] Docherty SJ Davis OS Haworth CM Plomin R Mill J DNA methylation profiling using bisulfite-based epityping of pooled genomic DNA Methods 2010 52 255 258 10.1016/j.ymeth.2010.06.017 20599507

[B14] Brooks AJ Waters MJ The growth hormone receptor: mechanism of activation and clinical implications Nat Rev Endocrinol 2010 6 515 525 10.1038/nrendo.2010.123 20664532

[B15] Banerjee-Basu S Packer A SFARI Gene: an evolving database for the autism research community Dis Model Mech 2010 3 133 135 10.1242/dmm.005439 20212079

[B16] Abrahams BS Geschwind DH Advances in autism genetics: on the threshold of a new neurobiology Nat Rev Genet 2008 9 341 355 10.1038/nrg2346 18414403 PMC2756414

[B17] Glessner JT Wang K Cai G Korvatska O Kim CE Wood S Zhang H Estes A Brune CW Bradfield JP Imielinski M Frackelton EC Reichert J Crawford EL Munson J Sleiman PM Chiavacci R Annaiah K Thomas K Hou C Glaberson W Flory J Otieno F Garris M Soorya L Klei L Piven J Meyer KJ Anagnostou E Sakurai T Autism genome-wide copy number variation reveals ubiquitin and neuronal genes Nature 2009 459 569 573 10.1038/nature07953 19404257 PMC2925224

[B18] Gupta AR State MW Recent advances in the genetics of autism Biol Psychiatry 2007 61 429 437 10.1016/j.biopsych.2006.06.020 16996486

[B19] Gillberg C Wahlstrom J Johansson R Tornblom M Albertsson-Wikland K Folic acid as an adjunct in the treatment of children with the autism fragile-X syndrome (AFRAX) Dev Med Child Neurol 1986 28 624 627 3536640 10.1111/j.1469-8749.1986.tb03905.x

[B20] Suren P Roth C Bresnahan M Haugen M Hornig M Hirtz D Lie KK Lipkin WI Magnus P Reichborn-Kjennerud T Schjolberg S Davey SG Oyen AS Susser E Stoltenberg C Association between maternal use of folic acid supplements and risk of autism spectrum disorders in children JAMA 2013 309 570 577 10.1001/jama.2012.155925 23403681 PMC3908544

[B21] Tamura T Picciano MF Folate and human reproduction Am J Clin Nutr 2006 83 993 1016 16685040 10.1093/ajcn/83.5.993

[B22] Stubbs G Litt M Lis E Jackson R Voth W Lindberg A Litt R Adenosine deaminase activity decreased in autism J Am Acad Child Psychiatry 1982 21 71 74 10.1097/00004583-198201000-00012 7096833

[B23] Bottini N De Luca D Saccucci P Fiumara A Elia M Porfirio MC Lucarelli P Curatolo P Autism: evidence of association with adenosine deaminase genetic polymorphism Neurogenetics 2001 3 111 113 10.1007/s100480000104 11354825

[B24] Hettinger JA Liu X Holden JJ The G22A polymorphism of the ADA gene and susceptibility to autism spectrum disorders J Autism Dev Disord 2008 38 14 19 10.1007/s10803-006-0354-0 17340203

[B25] Ozsahin H Arredondo-Vega FX Santisteban I Fuhrer H Tuchschmid P Jochum W Aguzzi A Lederman HM Fleischman A Winkelstein JA Seger RA Hershfield MS Adenosine deaminase deficiency in adults Blood 1997 89 2849 2855 9108404

[B26] Hirschhorn R Adenosine deaminase deficiency Immunodefic Rev 1990 2 175 198 2078332

[B27] Endo M Doi R Nishita M Minami Y Ror family receptor tyrosine kinases regulate the maintenance of neural progenitor cells in the developing neocortex J Cell Sci 2012 125 2017 2029 10.1242/jcs.097782 22328498

[B28] Tokuraku K Okuyama S Matsushima K Ikezu T Kotani S Distinct neuronal localization of microtubule-associated protein 4 in the mammalian brain Neurosci Lett 2010 484 143 147 10.1016/j.neulet.2010.08.038 20727942

[B29] Shah ZH O’Dell KM Miller SC An X Jacobs HT Metazoan nuclear genes for mitoribosomal protein S12 Gene 1997 204 55 62 10.1016/S0378-1119(97)00521-0 9434165

[B30] Dyachenko OV Schevchuk TV Kretzner L Buryanov YI Smith SS Human non-CG methylation: are human stem cells plant-like? Epigenetics 2010 5 569 572 10.4161/epi.5.7.12702 20647766 PMC3037444

[B31] Yan J Zierath JR Barres R Evidence for non-CpG methylation in mammals Exp Cell Res 2011 317 2555 2561 10.1016/j.yexcr.2011.08.019 21925168

[B32] Lister R Pelizzola M Dowen RH Hawkins RD Hon G Tonti-Filippini J Nery JR Lee L Ye Z Ngo QM Edsall L Antosiewicz-Bourget J Stewart R Ruotti V Millar AH Thomson JA Ren B Ecker JR Human DNA methylomes at base resolution show widespread epigenomic differences Nature 2009 462 315 322 10.1038/nature08514 19829295 PMC2857523

[B33] Fuso A Nicolia V Pasqualato A Fiorenza MT Cavallaro RA Scarpa S Changes in Presenilin 1 gene methylation pattern in diet-induced B vitamin deficiency Neurobiol Aging 2011 32 187 199 10.1016/j.neurobiolaging.2009.02.013 19329227

[B34] Inoue S Oishi M Effects of methylation of non-CpG sequence in the promoter region on the expression of human synaptotagmin XI (syt11) Gene 2005 348 123 134 15777718 10.1016/j.gene.2004.12.044

[B35] Vu TH Li T Nguyen D Nguyen BT Yao XM Hu JF Hoffman AR Symmetric and asymmetric DNA methylation in the human IGF2-H19 imprinted region Genomics 2000 64 132 143 10.1006/geno.1999.6094 10729220

[B36] Malone CS Miner MD Doerr JR Jackson JP Jacobsen SE Wall R Teitell M CmC(A/T)GG DNA methylation in mature B cell lymphoma gene silencing Proc Natl Acad Sci U S A 2001 98 10404 10409 10.1073/pnas.181206898 11504918 PMC56973

[B37] Teng C Gladwell W Raphiou I Liu E Methylation and expression of the lactoferrin gene in human tissues and cancer cells Biometals 2004 17 317 323 15222484 10.1023/b:biom.0000027711.13818.8a

[B38] Haines TR Rodenhiser DI Ainsworth PJ Allele-specific non-CpG methylation of the Nf1 gene during early mouse development Dev Biol 2001 240 585 598 10.1006/dbio.2001.0504 11784085

[B39] Suzuki MM Bird A DNA methylation landscapes: provocative insights from epigenomics Nat Rev Genet 2008 9 465 476 10.1038/nrg2341 18463664

[B40] Ball MP Li JB Gao Y Lee JH LeProust EM Park IH Xie B Daley GQ Church GM Targeted and genome-scale strategies reveal gene-body methylation signatures in human cells Nat Biotechnol 2009 27 361 368 10.1038/nbt.1533 19329998 PMC3566772

[B41] Hellman A Chess A Gene body-specific methylation on the active X chromosome Science 2007 315 1141 1143 10.1126/science.1136352 17322062

[B42] Rauch TA Wu X Zhong X Riggs AD Pfeifer GP A human B cell methylome at 100-base pair resolution Proc Natl Acad Sci USA 2009 106 671 678 10.1073/pnas.0812399106 19139413 PMC2621253

[B43] Oh JE Chambwe N Klein S Gal J Andrews S Gleason G Shaknovich R Melnick A Campagne F Toth M Differential gene body methylation and reduced expression of cell adhesion and neurotransmitter receptor genes in adverse maternal environment Transl Psychiatry 2013 3 e218 10.1038/tp.2012.130 23340501 PMC3566713

[B44] Moessner R Marshall CR Sutcliffe JS Skaug J Pinto D Vincent J Zwaigenbaum L Fernandez B Roberts W Szatmari P Scherer SW Contribution of SHANK3 mutations to autism spectrum disorder Am J Hum Genet 2007 81 1289 1297 10.1086/522590 17999366 PMC2276348

[B45] Oksenberg N Ahituv N The role of AUTS2 in neurodevelopment and human evolution Trends Genet 2013 29 600 608 10.1016/j.tig.2013.08.001 24008202 PMC3823538

[B46] Gallou-Kabani C Gabory A Tost J Karimi M Mayeur S Lesage J Boudadi E Gross MS Taurelle J Vige A Breton C Reusens B Remacle C Vieau D Ekstrom TJ Jais JP Junien C Sex- and diet-specific changes of imprinted gene expression and DNA methylation in mouse placenta under a high-fat diet PLoS One 2010 5 e14398 10.1371/journal.pone.0014398 21200436 PMC3006175

[B47] Gabory A Attig L Junien C Developmental programming and epigenetics Am J Clin Nutr 2011 94 1943S 1952S 10.3945/ajcn.110.000927 22049164

[B48] Gabory A Attig L Junien C Sexual dimorphism in environmental epigenetic programming Mol Cell Endocrinol 2009 304 8 18 10.1016/j.mce.2009.02.015 19433243

[B49] Flanagan DE Moore VM Godsland IF Cockington RA Robinson JS Phillips DI Fetal growth and the physiological control of glucose tolerance in adults: a minimal model analysis Am J Physiol Endocrinol Metab 2000 278 E700 E706 10751205 10.1152/ajpendo.2000.278.4.E700

[B50] Sugden MC Holness MJ Gender-specific programming of insulin secretion and action J Endocrinol 2002 175 757 767 10.1677/joe.0.1750757 12475386

[B51] Ozaki T Nishina H Hanson MA Poston L Dietary restriction in pregnant rats causes gender-related hypertension and vascular dysfunction in offspring J Physiol 2001 530 141 152 10.1111/j.1469-7793.2001.0141m.x 11136866 PMC2278385

[B52] Kwong WY Wild AE Roberts P Willis AC Fleming TP Maternal undernutrition during the preimplantation period of rat development causes blastocyst abnormalities and programming of postnatal hypertension Development 2000 127 4195 4202 10976051 10.1242/dev.127.19.4195

[B53] Woods LL Weeks DA Rasch R Programming of adult blood pressure by maternal protein restriction: role of nephrogenesis Kidney Int 2004 65 1339 1348 10.1111/j.1523-1755.2004.00511.x 15086473

[B54] Friso S Choi SW Girelli D Mason JB Dolnikowski GG Bagley PJ Olivieri O Jacques PF Rosenberg IH Corrocher R Selhub J A common mutation in the 5,10-methylenetetrahydrofolate reductase gene affects genomic DNA methylation through an interaction with folate status Proc Natl Acad Sci USA 2002 99 5606 5611 10.1073/pnas.062066299 11929966 PMC122817

[B55] Chen Z Schwahn BC Wu Q He X Rozen R Postnatal cerebellar defects in mice deficient in methylenetetrahydrofolate reductase Int J Dev Neurosci 2005 23 465 474 10.1016/j.ijdevneu.2005.05.007 15979267

[B56] Guintivano J Aryee MJ Kaminsky ZA A cell epigenotype specific model for the correction of brain cellular heterogeneity bias and its application to age, brain region and major depression Epigenetics 2013 8 290 302 10.4161/epi.23924 23426267 PMC3669121

[B57] Iwamoto K Bundo M Ueda J Oldham MC Ukai W Hashimoto E Saito T Geschwind DH Kato T Neurons show distinctive DNA methylation profile and higher interindividual variations compared with non-neurons Genome Res 2011 21 688 696 10.1101/gr.112755.110 21467265 PMC3083085

[B58] Ohgane J Yagi S Shiota K Epigenetics: the DNA methylation profile of tissue-dependent and differentially methylated regions in cells Placenta 2008 29 Suppl A S29 S35 18031808 10.1016/j.placenta.2007.09.011

[B59] Tichopad A Dilger M Schwarz G Pfaffl MW Standardized determination of real-time PCR efficiency from a single reaction set-up Nucleic Acids Res 2003 31 e122 10.1093/nar/gng122 14530455 PMC219490

[B60] Amaral DG Schumann CM Nordahl CW Neuroanatomy of autism Trends Neurosci 2008 31 137 145 10.1016/j.tins.2007.12.005 18258309

[B61] Courchesne E Pierce K Why the frontal cortex in autism might be talking only to itself: local over-connectivity but long-distance disconnection Curr Opin Neurobiol 2005 15 225 230 10.1016/j.conb.2005.03.001 15831407

