# Supplementary material for: Single-base resolution of mouse offspring brain methylome reveals epigenome modifications caused by gestational folic acid
Source: Epigenetics Chromatin. 2014 Feb 3;7:3. doi: 10.1186/1756-8935-7-3 (PMC3928622; doi:10.1186/1756-8935-7-3)
Supplement: Additional file 1: Table S1 — Descriptive statistics of the mapping of methylation profile of the cerebral hemispheres of offspring from low maternal folic acid (LMFA) and high maternal folic acid (HMFA) diets. [file 1756-8935-7-3-S1.pdf]

Table-S1: Descriptive statistics of the mapping of methylation profile of pups cerebral hemispheres from mothers supplemented with FA at 0.4 and 4 mg/kg of diet.

Cerebral hemispheres	Total Read	Mapped Read	Mapping Ratio	Unique CpG	CpG Coverage (X)	Bisufite Conv Rate
Male-0.4	30,727,284	14,503,542	47.20%	4,525,416	12	98.28%
Male-4	28,503,239	12,951,562	45.44%	4,768,860	10	98.25%
Female-0.4	32,518,299	16,960,824	52.16%	4,556,488	14	98.44%
Female-4	34,945,143	16,219,404	46.41%	4,264,472	14	98.86%
