# Supplementary figures and images for: Single-base resolution of mouse offspring brain methylome reveals epigenome modifications caused by gestational folic acid
Source: Epigenetics Chromatin. 2014 Feb 3;7:3. doi: 10.1186/1756-8935-7-3 (PMC3928622; doi:10.1186/1756-8935-7-3)

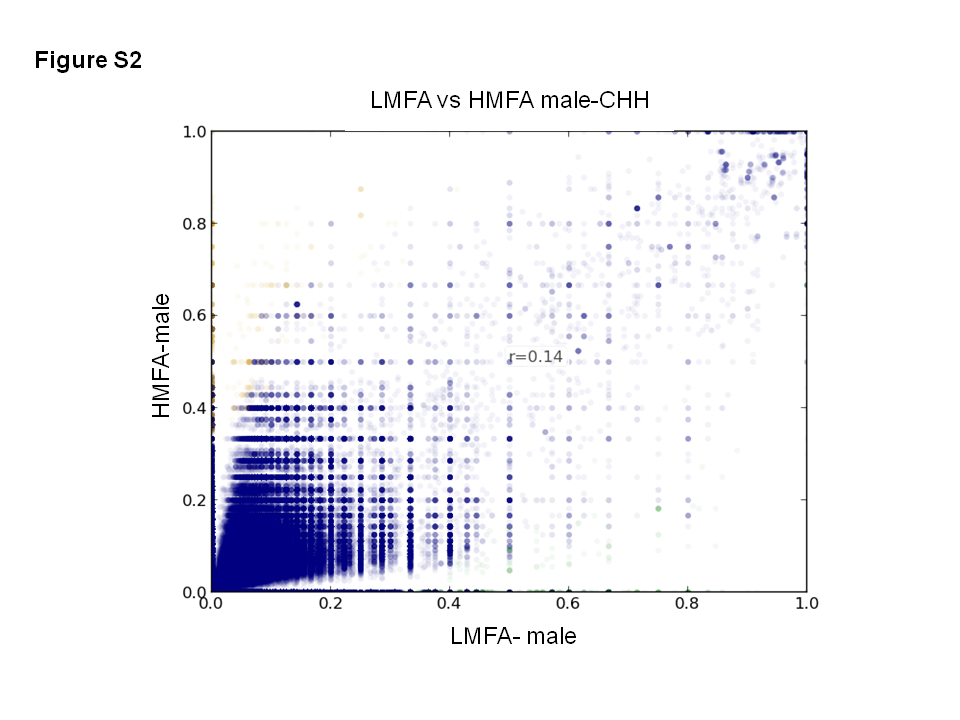

Supplement: Additional file 6: Figures S1-S6 — Scatter plot representing the distribution of the methylation ratio for corresponding sites of low maternal folic acid (LMFA) versus high maternal folic acid (HMFA). Pearson’s correlation coefficient is denoted in the center of each scatter plot. [file 1756-8935-7-3-S6.zip › Figure S2.tif]

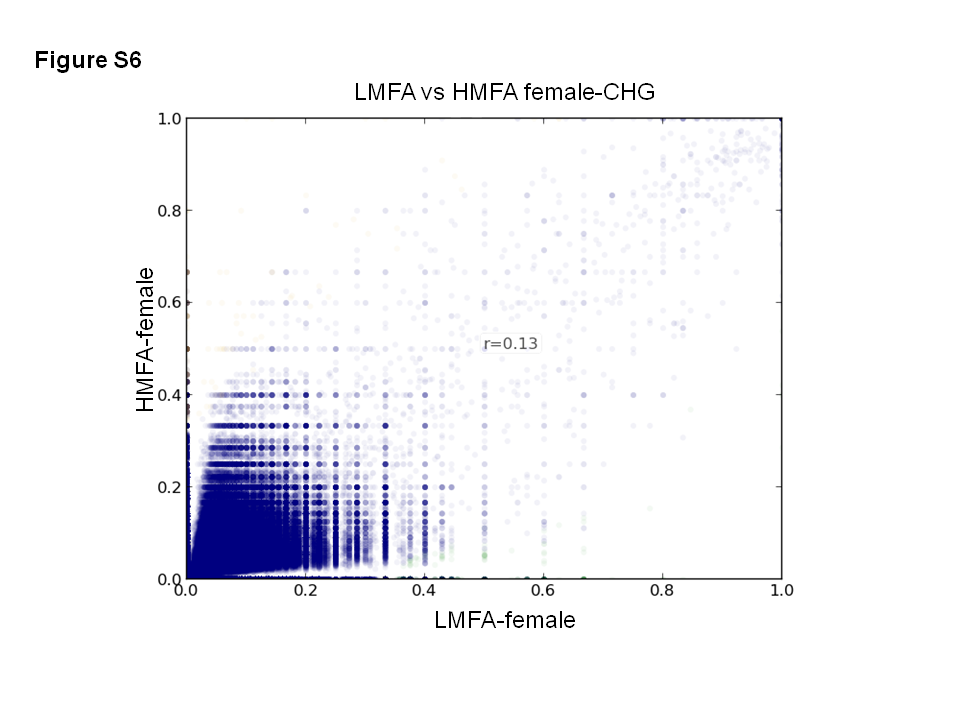

Supplement: Additional file 6: Figures S1-S6 — Scatter plot representing the distribution of the methylation ratio for corresponding sites of low maternal folic acid (LMFA) versus high maternal folic acid (HMFA). Pearson’s correlation coefficient is denoted in the center of each scatter plot. [file 1756-8935-7-3-S6.zip › Figure S6.tif]

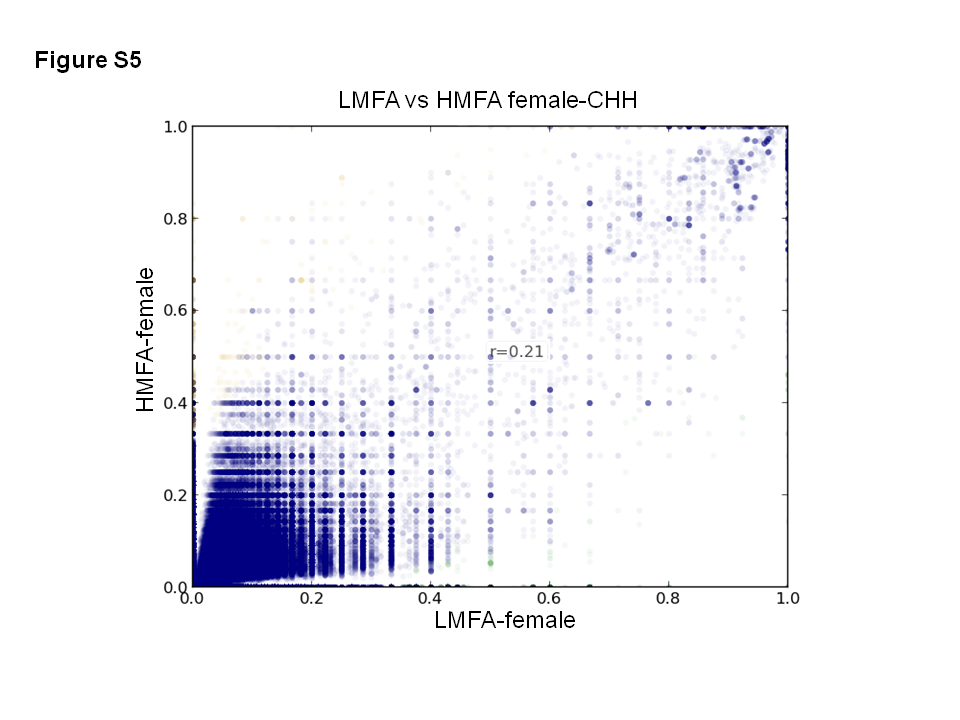

Supplement: Additional file 6: Figures S1-S6 — Scatter plot representing the distribution of the methylation ratio for corresponding sites of low maternal folic acid (LMFA) versus high maternal folic acid (HMFA). Pearson’s correlation coefficient is denoted in the center of each scatter plot. [file 1756-8935-7-3-S6.zip › Figure S5.tif]

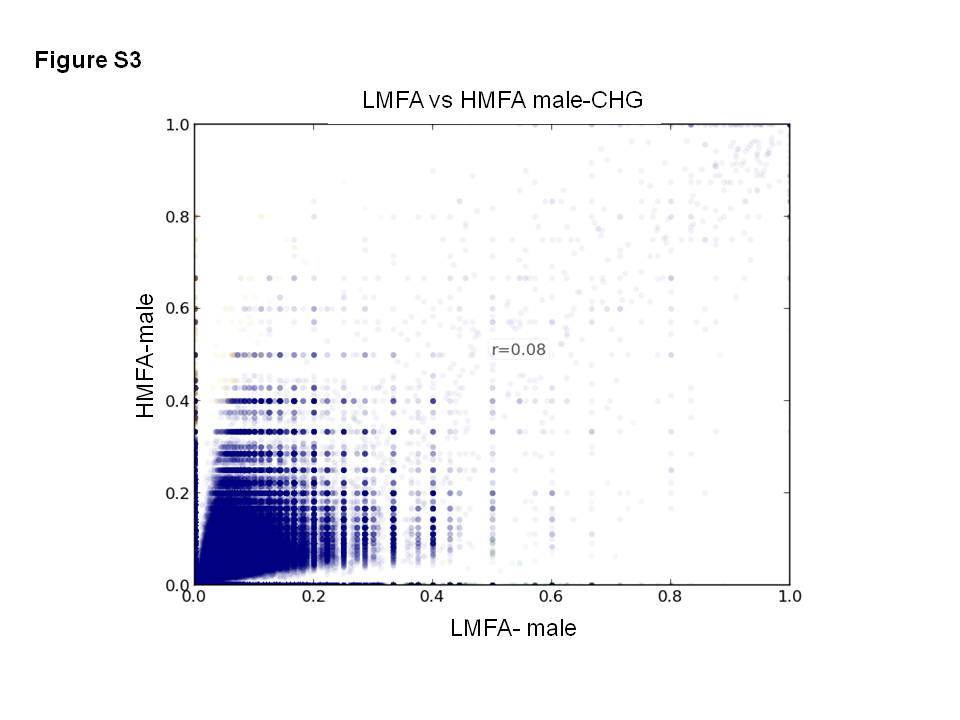

Supplement: Additional file 6: Figures S1-S6 — Scatter plot representing the distribution of the methylation ratio for corresponding sites of low maternal folic acid (LMFA) versus high maternal folic acid (HMFA). Pearson’s correlation coefficient is denoted in the center of each scatter plot. [file 1756-8935-7-3-S6.zip › Figure S3.tif]

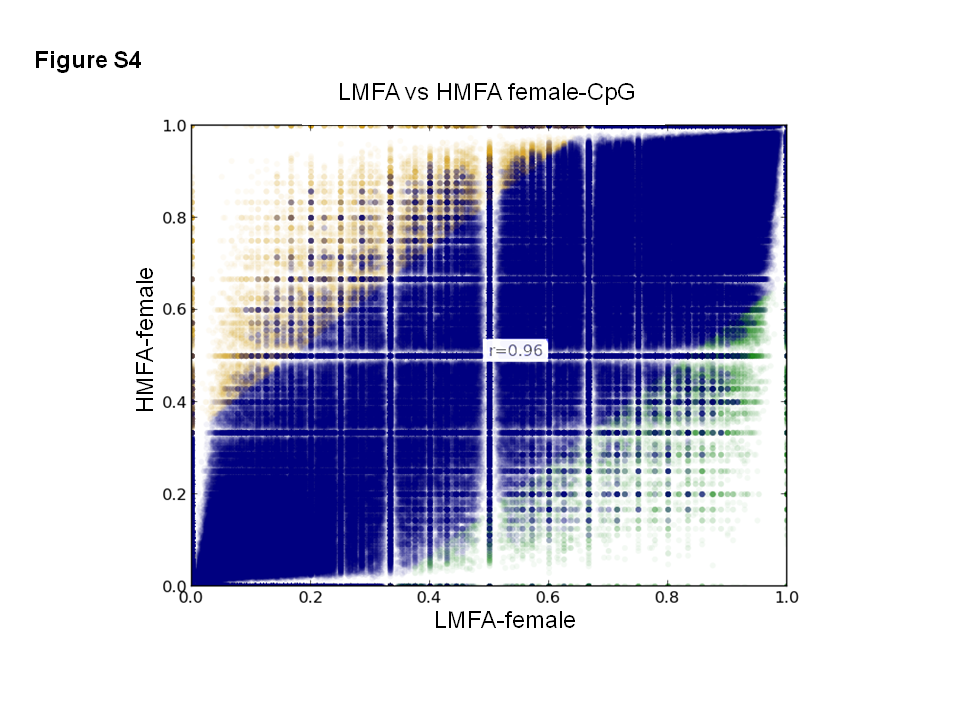

Supplement: Additional file 6: Figures S1-S6 — Scatter plot representing the distribution of the methylation ratio for corresponding sites of low maternal folic acid (LMFA) versus high maternal folic acid (HMFA). Pearson’s correlation coefficient is denoted in the center of each scatter plot. [file 1756-8935-7-3-S6.zip › Figure S4.tif]

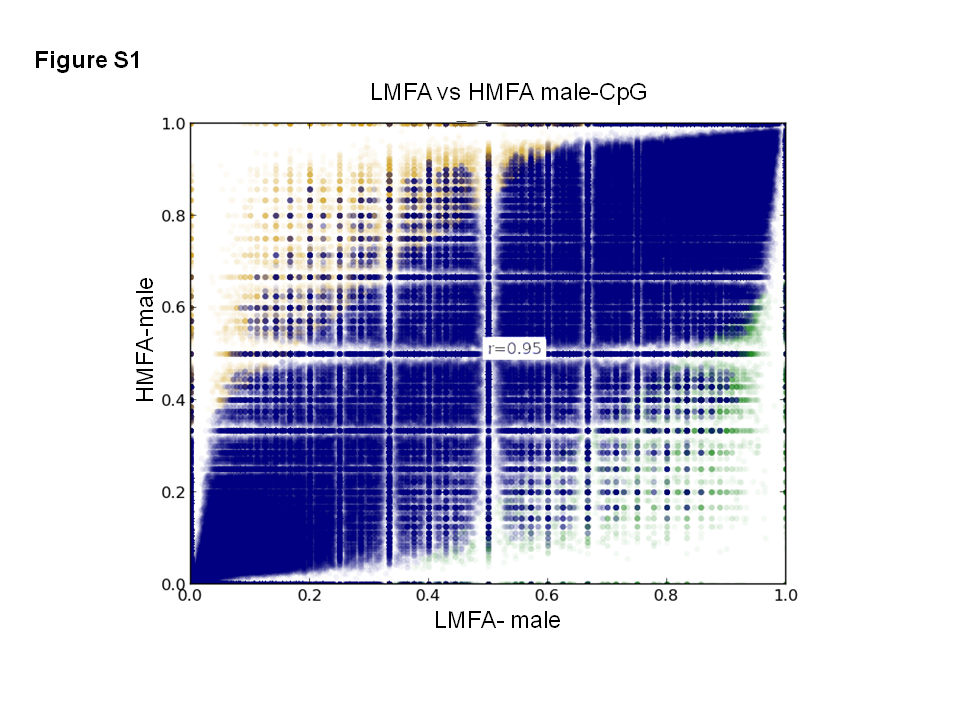

Supplement: Additional file 6: Figures S1-S6 — Scatter plot representing the distribution of the methylation ratio for corresponding sites of low maternal folic acid (LMFA) versus high maternal folic acid (HMFA). Pearson’s correlation coefficient is denoted in the center of each scatter plot. [file 1756-8935-7-3-S6.zip › Figure S1.tif]

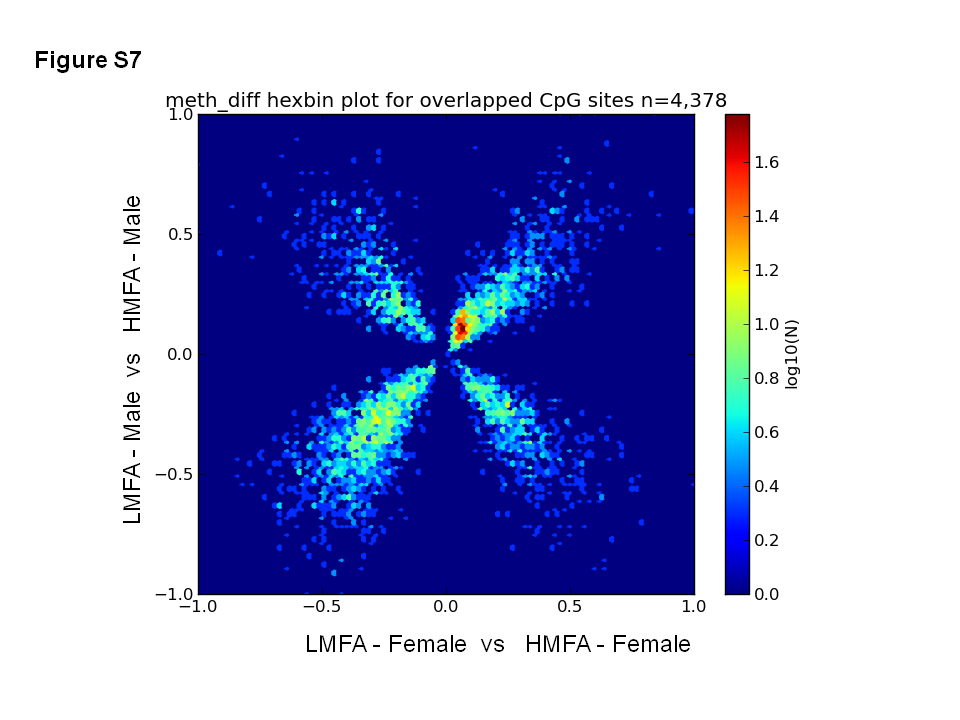

Supplement: Additional file 7: Figures S7-S9 — Hexbin plot representing the overlapped sites in CpG (n = 4,378), CHG (n = 71), and CHH (n = 149) regions between male and female pups from high maternal folic acid (HMFA) in comparison with low maternal folic acid (LMFA) from total significant (P <0.05) differential methylation sites. Each dot in hexbin plot is one of the overlapped sites. The colors blue, green, yellow and red represent the dot density from lower to higher order in accordance with the prevalence of the overlapping sites. [file 1756-8935-7-3-S7.zip › 13072_6071034771062435_MOESM7_ESM/6071034771062435_add7/Figure S7.tif]

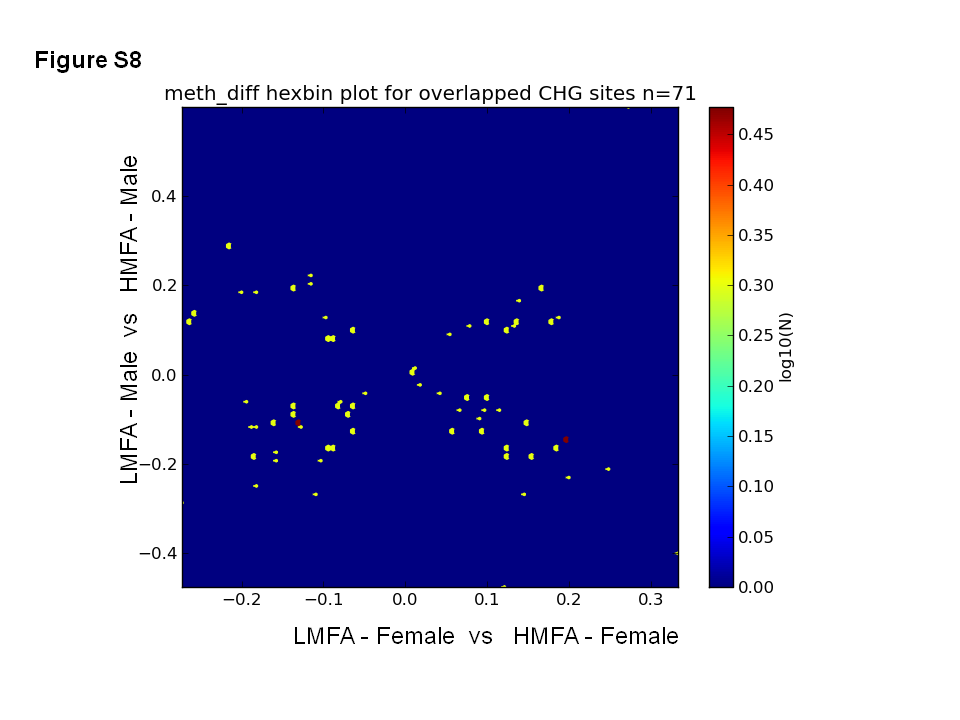

Supplement: Additional file 7: Figures S7-S9 — Hexbin plot representing the overlapped sites in CpG (n = 4,378), CHG (n = 71), and CHH (n = 149) regions between male and female pups from high maternal folic acid (HMFA) in comparison with low maternal folic acid (LMFA) from total significant (P <0.05) differential methylation sites. Each dot in hexbin plot is one of the overlapped sites. The colors blue, green, yellow and red represent the dot density from lower to higher order in accordance with the prevalence of the overlapping sites. [file 1756-8935-7-3-S7.zip › 13072_6071034771062435_MOESM7_ESM/6071034771062435_add7/Figure S8.tif]

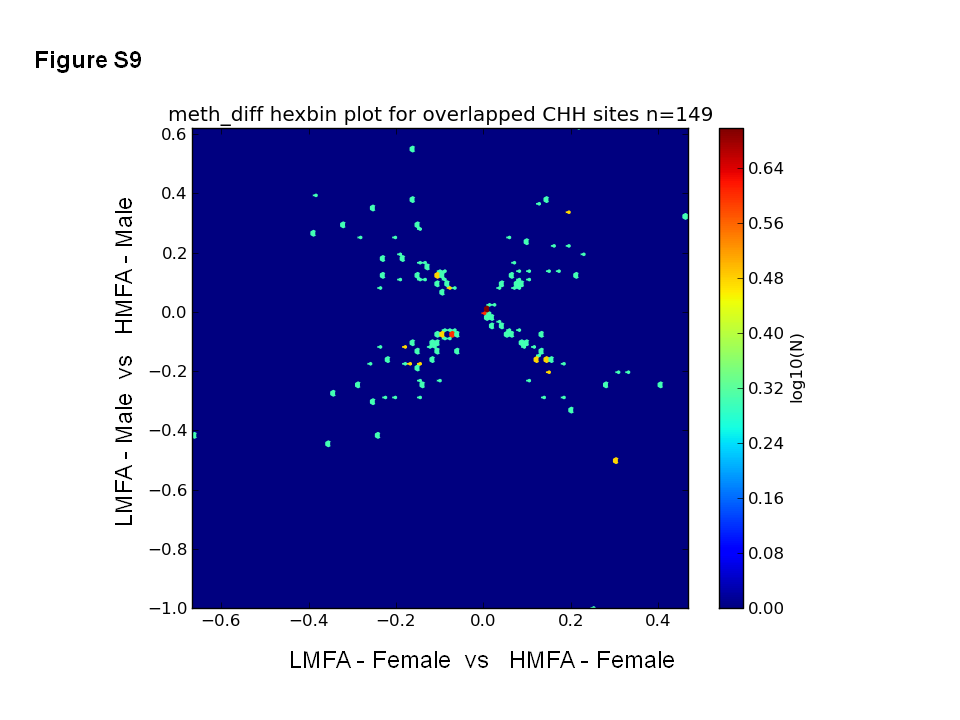

Supplement: Additional file 7: Figures S7-S9 — Hexbin plot representing the overlapped sites in CpG (n = 4,378), CHG (n = 71), and CHH (n = 149) regions between male and female pups from high maternal folic acid (HMFA) in comparison with low maternal folic acid (LMFA) from total significant (P <0.05) differential methylation sites. Each dot in hexbin plot is one of the overlapped sites. The colors blue, green, yellow and red represent the dot density from lower to higher order in accordance with the prevalence of the overlapping sites. [file 1756-8935-7-3-S7.zip › 13072_6071034771062435_MOESM7_ESM/6071034771062435_add7/Figure S9.tif]

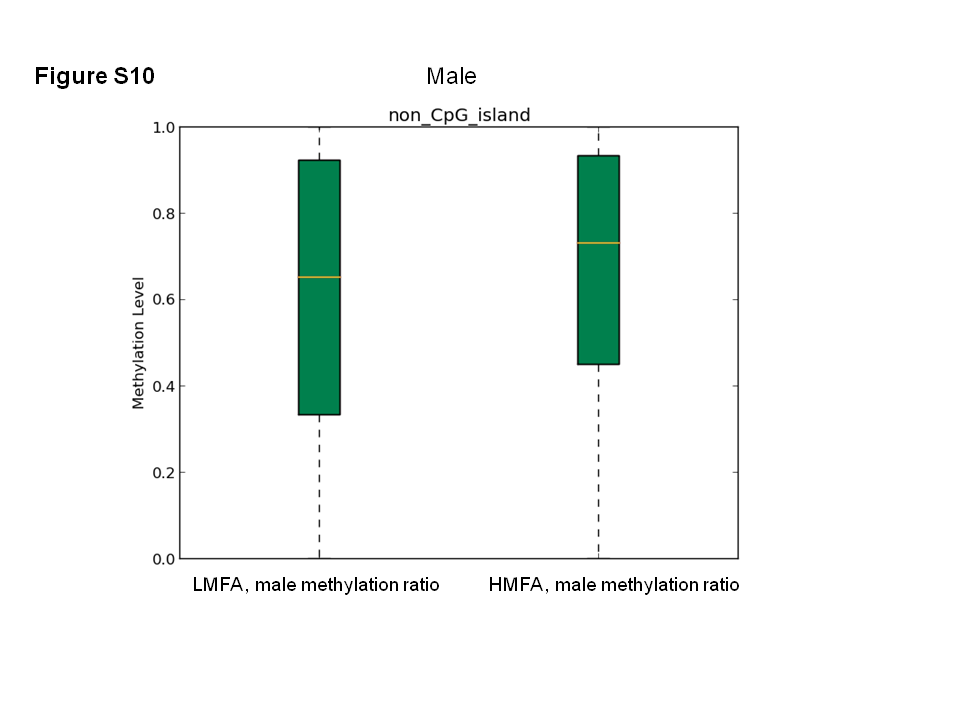

Supplement: Additional file 8: Figures S10, S11 — Box plot illustrating the methylation levels across non-CpG islands of the cerebral hemispheres from male pups (S10) having low maternal folic acid (LMFA) (n = 36,319 and median = 0.65) and high maternal folic acid (HMFA) (n = 36,319 and median = 0.73) and from female pups (S11) having LMFA (n = 48,438 and median = 0.66) and HMFA (n = 48,438 and median = 0.69) as assessed by reduced representation bisulfite sequencing (RRBS). Boxes are 25th and 75th quartiles; horizontal yellow bar in the middle represents the median DNA methylation value. Whisker indicates the 5th and 95th percentiles. [file 1756-8935-7-3-S8.zip › 13072_6071034771062435_MOESM8_ESM/6071034771062435_add8/Figure S10.tif]

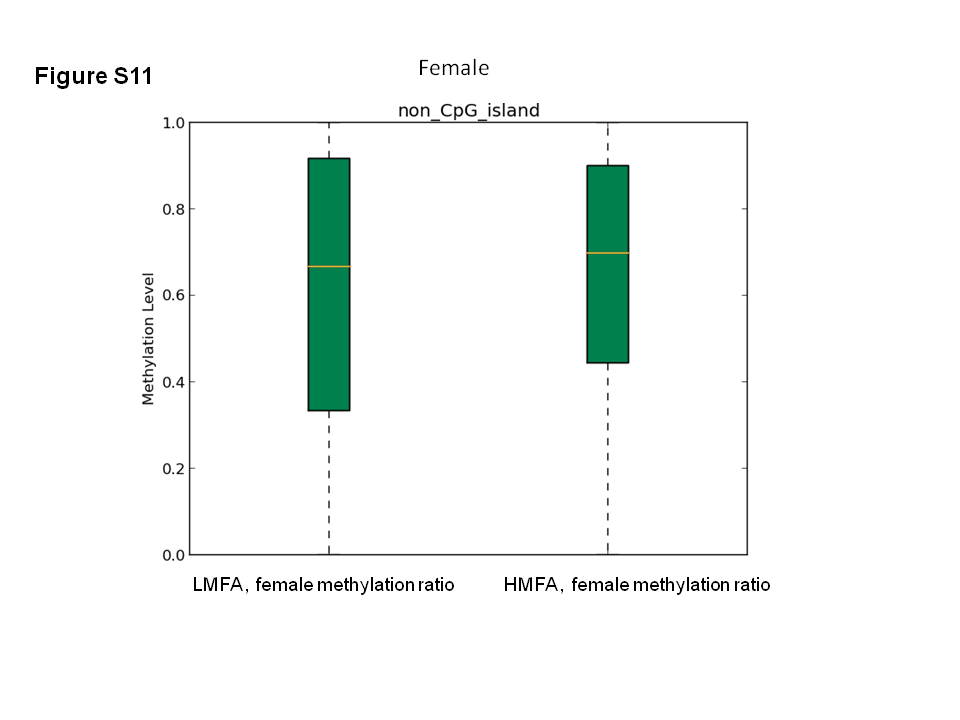

Supplement: Additional file 8: Figures S10, S11 — Box plot illustrating the methylation levels across non-CpG islands of the cerebral hemispheres from male pups (S10) having low maternal folic acid (LMFA) (n = 36,319 and median = 0.65) and high maternal folic acid (HMFA) (n = 36,319 and median = 0.73) and from female pups (S11) having LMFA (n = 48,438 and median = 0.66) and HMFA (n = 48,438 and median = 0.69) as assessed by reduced representation bisulfite sequencing (RRBS). Boxes are 25th and 75th quartiles; horizontal yellow bar in the middle represents the median DNA methylation value. Whisker indicates the 5th and 95th percentiles. [file 1756-8935-7-3-S8.zip › 13072_6071034771062435_MOESM8_ESM/6071034771062435_add8/Figure S11.tif]

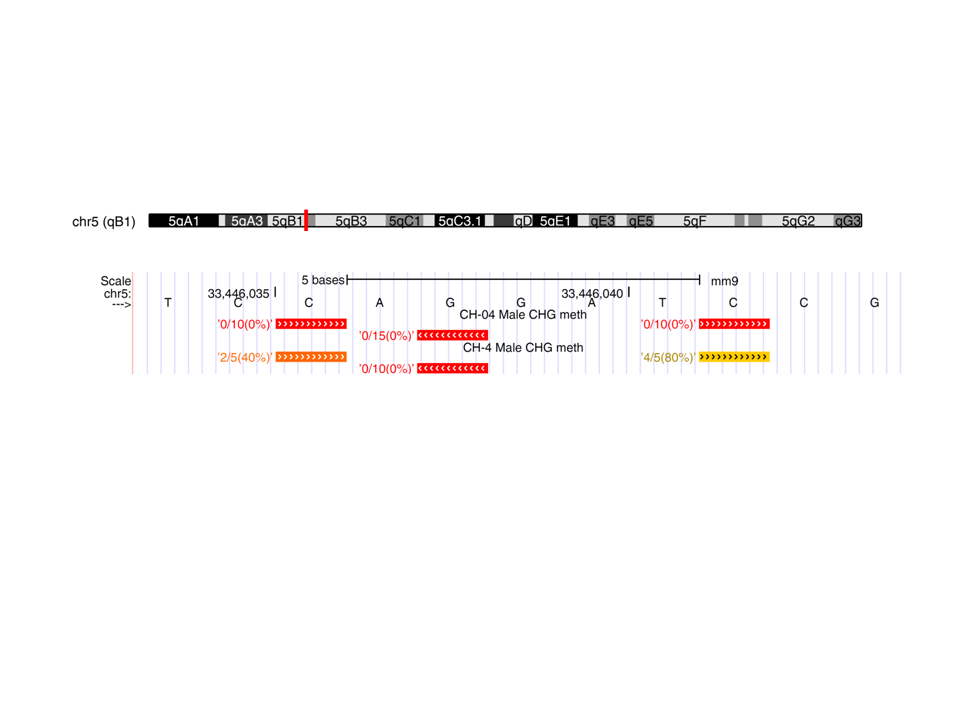

Supplement: Additional file 10: Figure S12 — A representative figure of the data uploaded in the University of California at Santa Cruz (UCSC) Genome Browser. Comparison of DNA methylation patterns in the offspring’s cerebral hemisphere from low maternal folic acid (LMFA) and high maternal folic acid (HMFA). Example of the Slc5a1 gene in chromosome 5 that was differentially methylated in the CHG contexts of promoter region of male offspring. Yellow color bars indicate gain of methylation. [file 1756-8935-7-3-S10.tif]

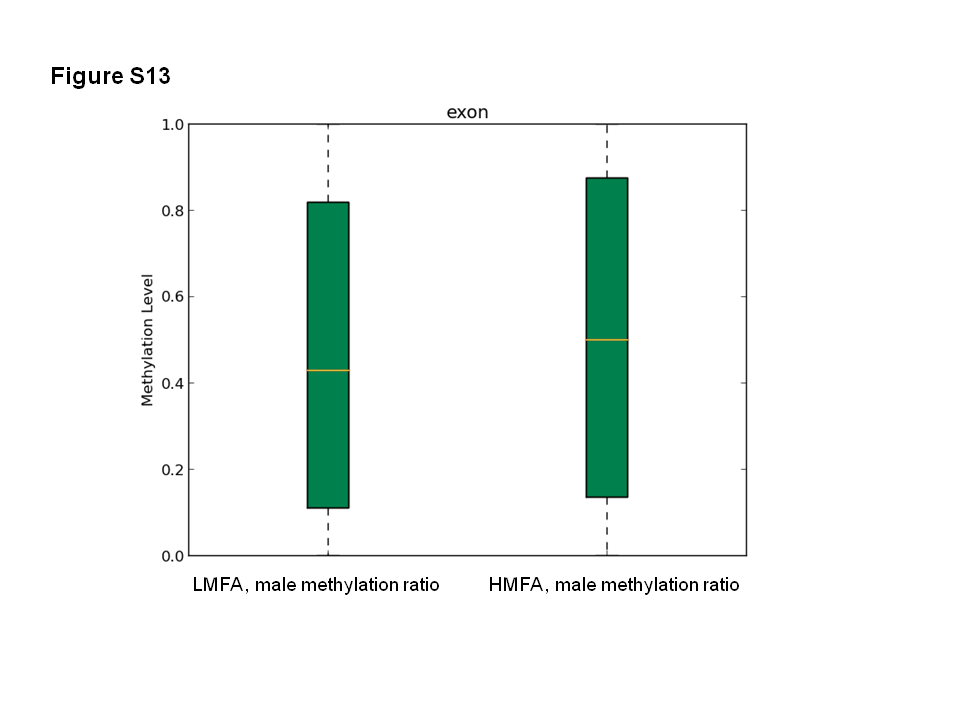

Supplement: Additional file 11: Figures S13, S14 — Box plot illustrating the methylation levels across exons of the cerebral hemispheres from male pups (S13) having low maternal folic acid (LMFA) (n = 8,136 and median = 0.42) and high maternal folic acid (HMFA) (n = 8,136 and median = 0.5) and from female pups (S14) having LMFA (n = 10,335 and median = 0.44) and HMFA (n = 10,335 and median = 0.5) as assessed by reduced representation bisulfite sequencing (RRBS). Boxes are 25th and 75th quartiles; horizontal yellow bar in the middle represents the median DNA methylation value. Whisker indicates the 5th and 95th percentiles. [file 1756-8935-7-3-S11.zip › Figure S13.tif]

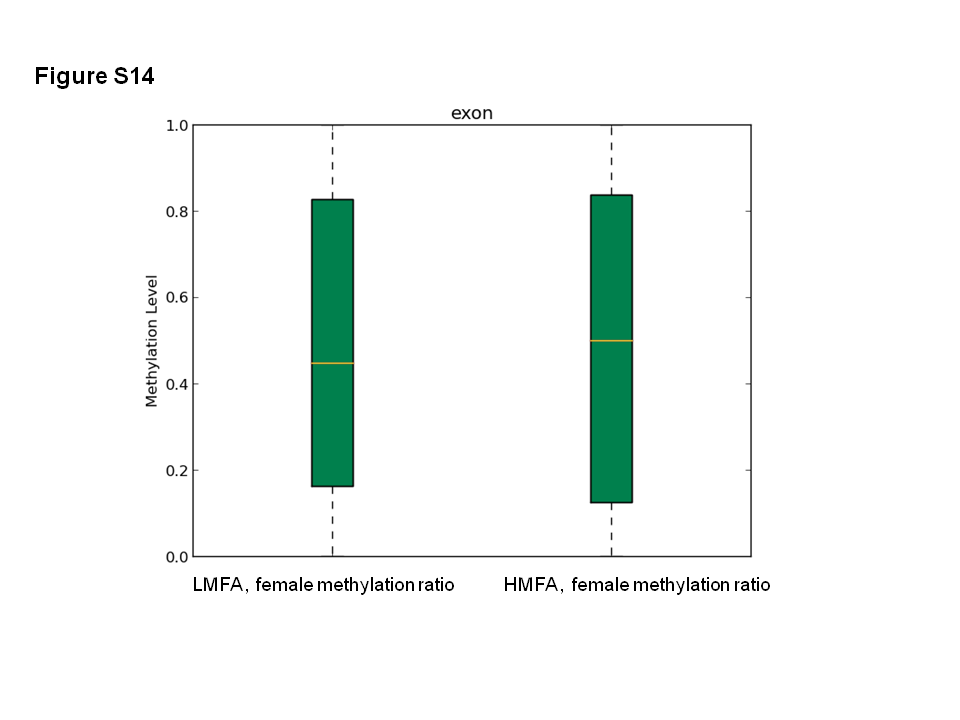

Supplement: Additional file 11: Figures S13, S14 — Box plot illustrating the methylation levels across exons of the cerebral hemispheres from male pups (S13) having low maternal folic acid (LMFA) (n = 8,136 and median = 0.42) and high maternal folic acid (HMFA) (n = 8,136 and median = 0.5) and from female pups (S14) having LMFA (n = 10,335 and median = 0.44) and HMFA (n = 10,335 and median = 0.5) as assessed by reduced representation bisulfite sequencing (RRBS). Boxes are 25th and 75th quartiles; horizontal yellow bar in the middle represents the median DNA methylation value. Whisker indicates the 5th and 95th percentiles. [file 1756-8935-7-3-S11.zip › Figure S14.tif]

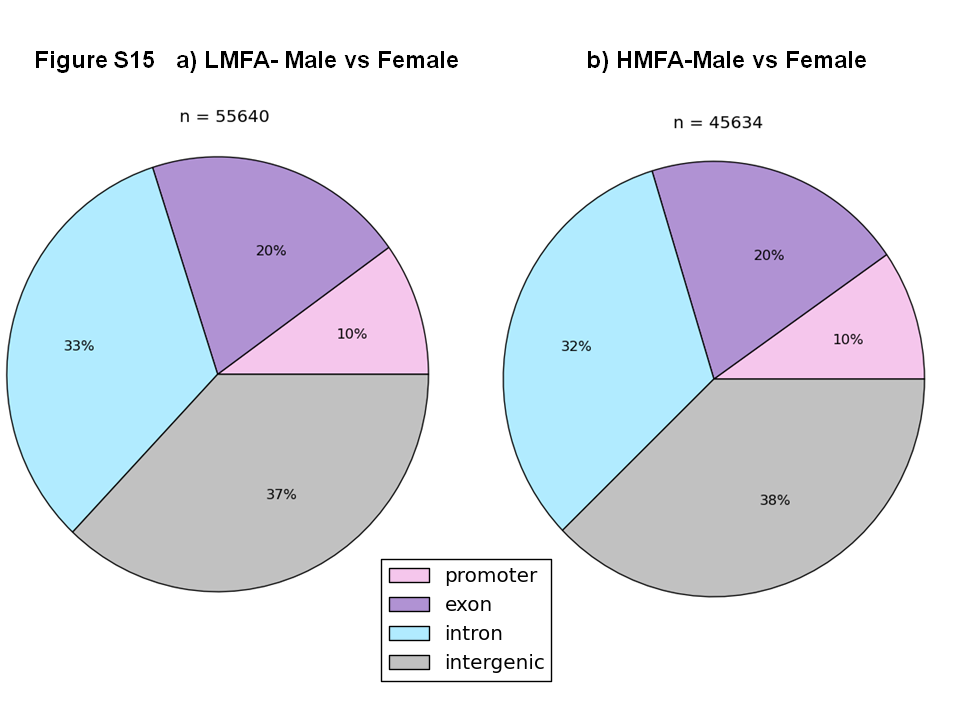

Supplement: Additional file 12: Figure S15 — Distribution of differentially methylated sites in CpG island sequences between males and females from (a) low maternal folic acid (LMFA) and (b) high maternal folic acid (HMFA). [file 1756-8935-7-3-S12.tiff]

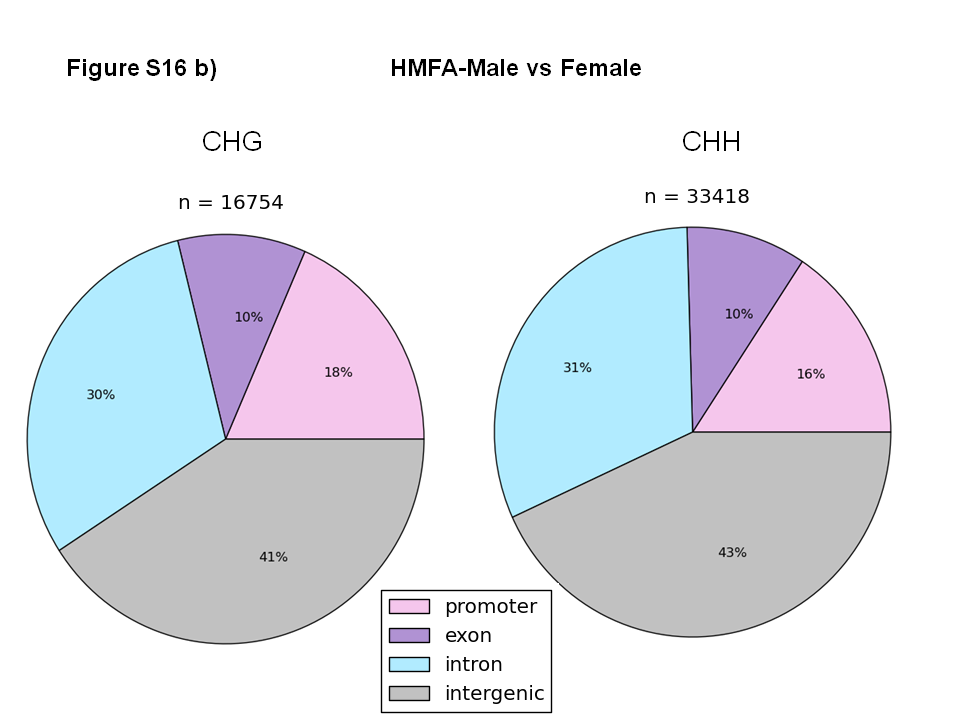

Supplement: Additional file 13: Figure S16 — Distribution of differentially methylated sites in non CpG island sequences between males and females from (a) low maternal folic acid (LMFA) and (b) high maternal folic acid (HMFA). [file 1756-8935-7-3-S13.zip › Figure S16b.tif]

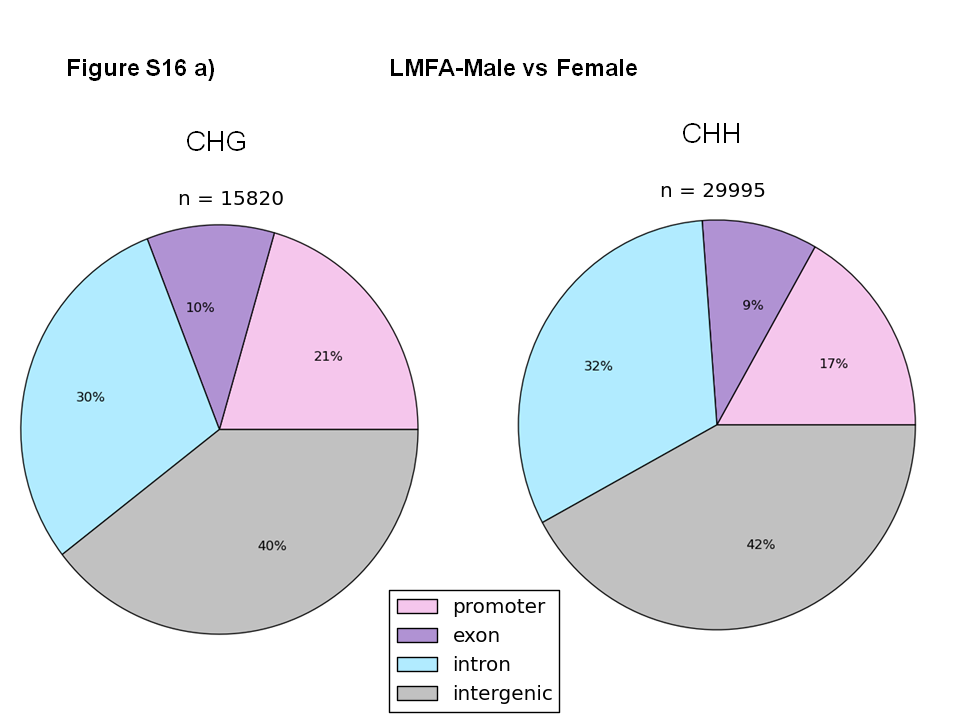

Supplement: Additional file 13: Figure S16 — Distribution of differentially methylated sites in non CpG island sequences between males and females from (a) low maternal folic acid (LMFA) and (b) high maternal folic acid (HMFA). [file 1756-8935-7-3-S13.zip › Figure S16a.tif]

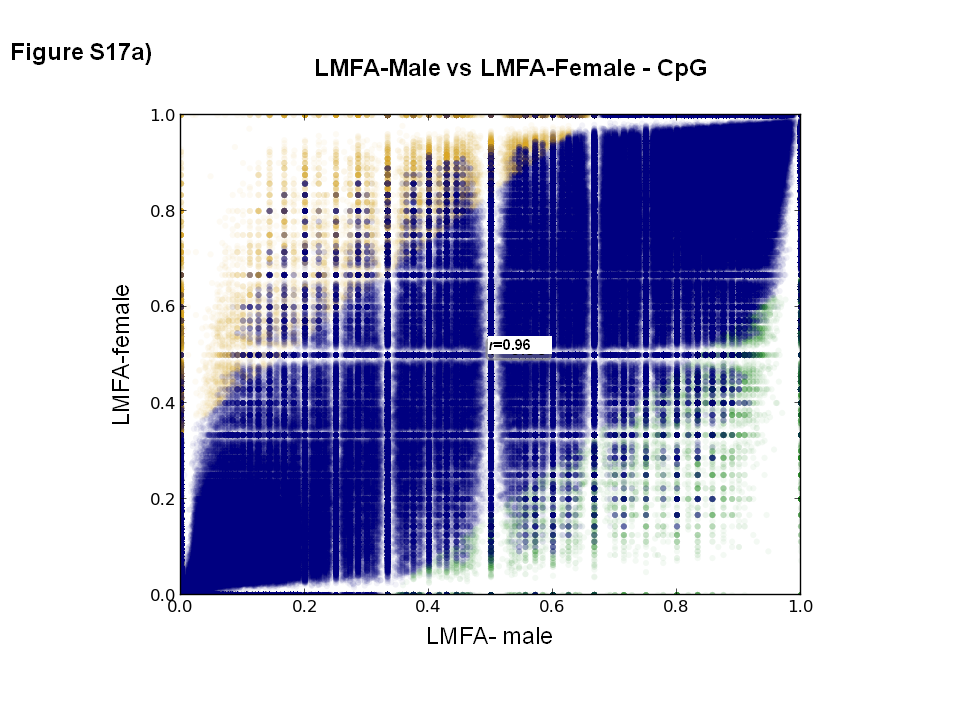

Supplement: Additional file 18: Figures S17 (a, b, c) — Scatter plot representing the distribution of the methylation ratio for corresponding sites of low maternal folic acid (LMFA) male versus LMFA female in CpG/CHG/CHH regions. Pearson’s correlation coefficient is denoted in the center of each scatter plot. [file 1756-8935-7-3-S18.zip › 13072_6071034771062435_MOESM18_ESM/6071034771062435_add18/Figure S17a.tif]

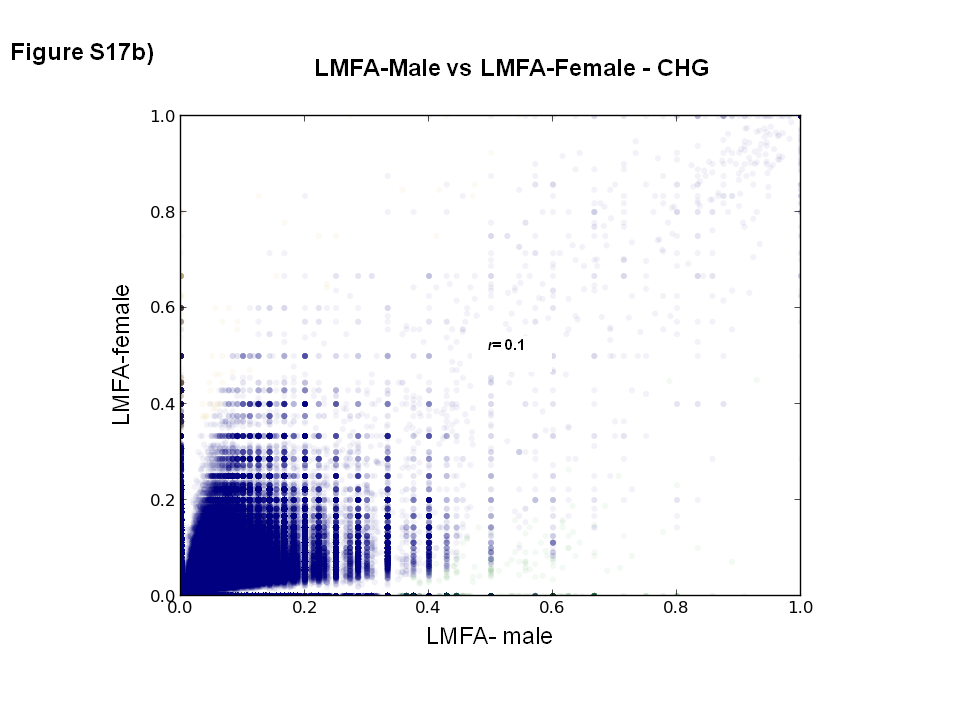

Supplement: Additional file 18: Figures S17 (a, b, c) — Scatter plot representing the distribution of the methylation ratio for corresponding sites of low maternal folic acid (LMFA) male versus LMFA female in CpG/CHG/CHH regions. Pearson’s correlation coefficient is denoted in the center of each scatter plot. [file 1756-8935-7-3-S18.zip › 13072_6071034771062435_MOESM18_ESM/6071034771062435_add18/Figure S17b.tif]

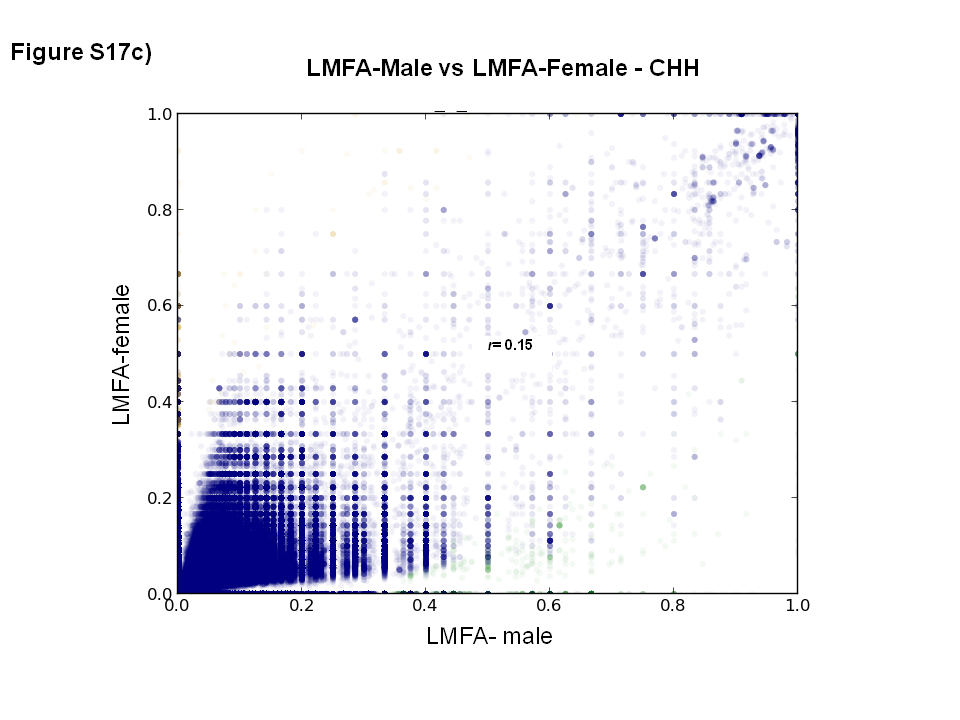

Supplement: Additional file 18: Figures S17 (a, b, c) — Scatter plot representing the distribution of the methylation ratio for corresponding sites of low maternal folic acid (LMFA) male versus LMFA female in CpG/CHG/CHH regions. Pearson’s correlation coefficient is denoted in the center of each scatter plot. [file 1756-8935-7-3-S18.zip › 13072_6071034771062435_MOESM18_ESM/6071034771062435_add18/Figure S17c.tif]

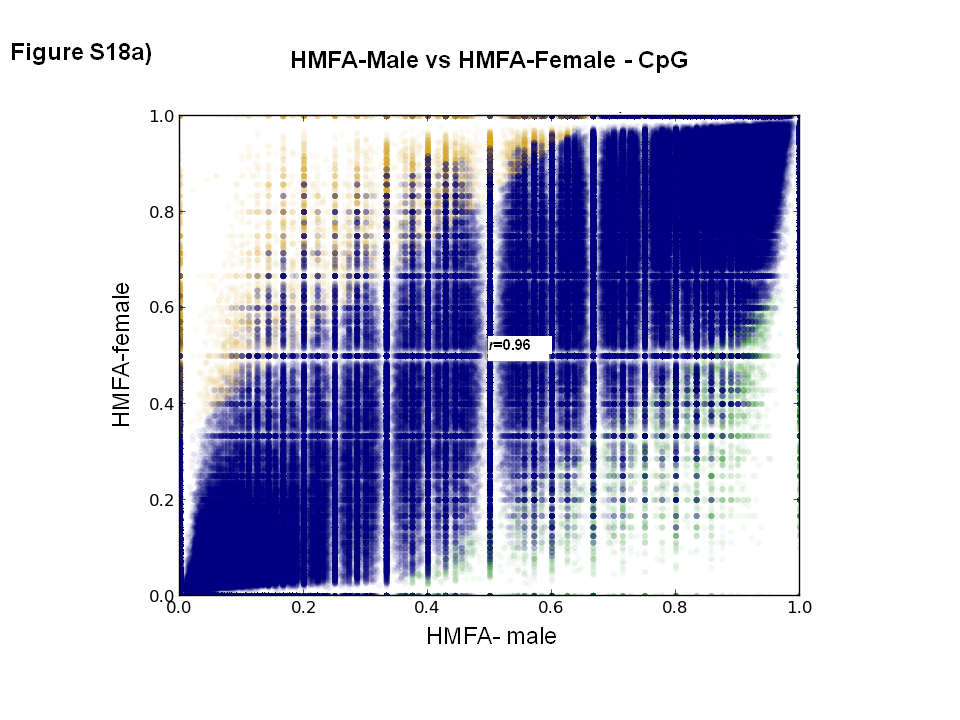

Supplement: Additional file 19: Figures S18 (a, b, c) — Scatter plot representing the distribution of the methylation ratio for corresponding sites of high maternal folic acid (HMFA) male versus HMFA female in CpG/CHG/CHH regions. Pearson’s correlation coefficient is denoted in the center of each scatter plot. [file 1756-8935-7-3-S19.zip › 13072_6071034771062435_MOESM19_ESM/6071034771062435_add19/Figure S18a.tif]

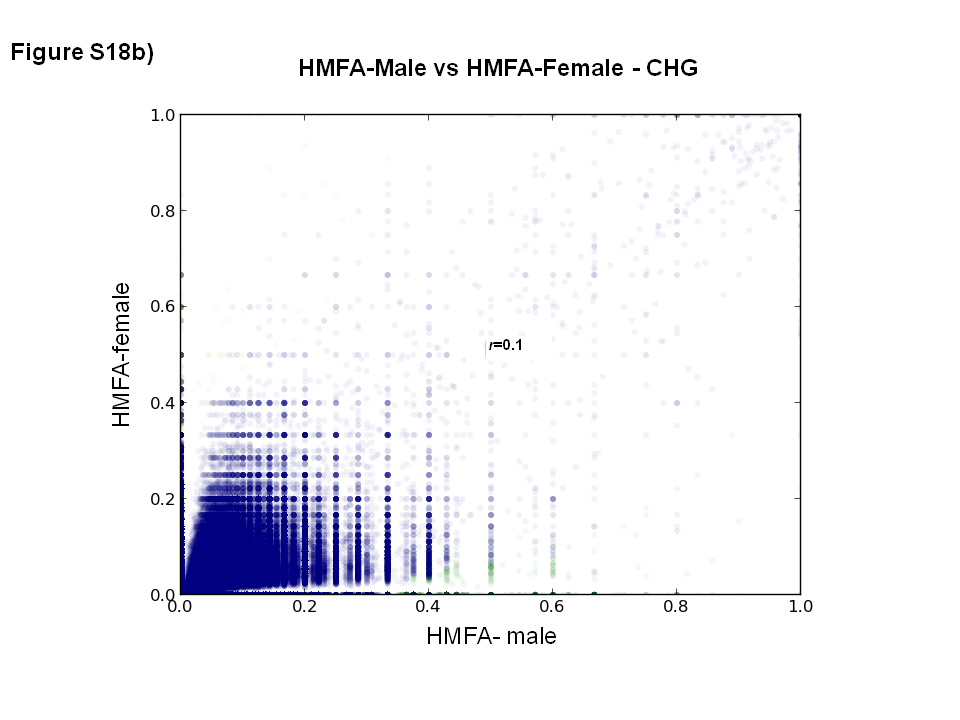

Supplement: Additional file 19: Figures S18 (a, b, c) — Scatter plot representing the distribution of the methylation ratio for corresponding sites of high maternal folic acid (HMFA) male versus HMFA female in CpG/CHG/CHH regions. Pearson’s correlation coefficient is denoted in the center of each scatter plot. [file 1756-8935-7-3-S19.zip › 13072_6071034771062435_MOESM19_ESM/6071034771062435_add19/Figure S18b.tif]

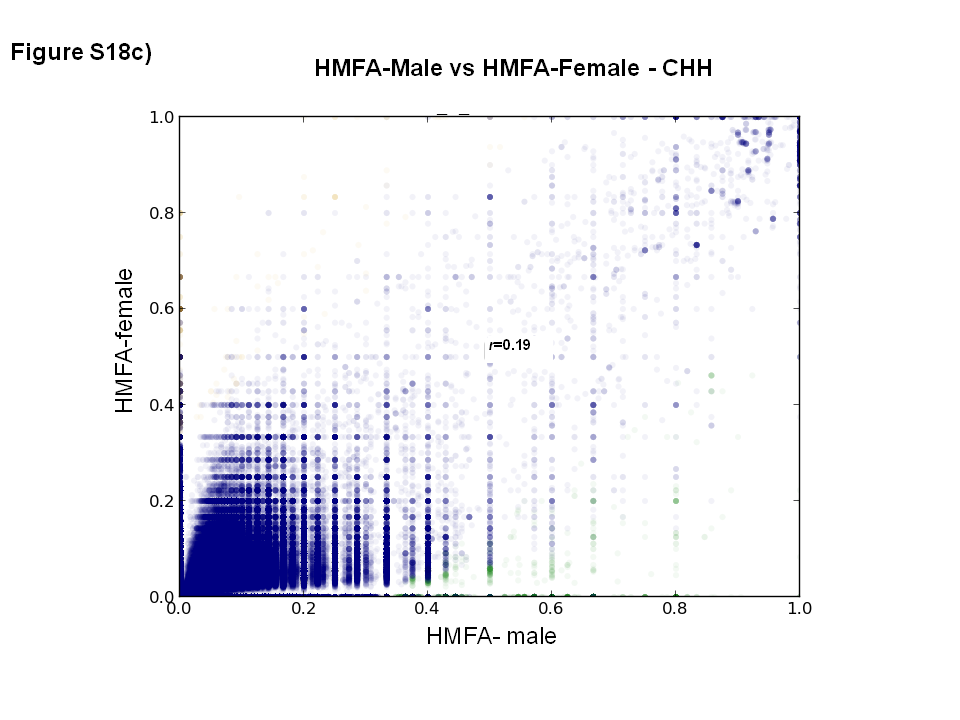

Supplement: Additional file 19: Figures S18 (a, b, c) — Scatter plot representing the distribution of the methylation ratio for corresponding sites of high maternal folic acid (HMFA) male versus HMFA female in CpG/CHG/CHH regions. Pearson’s correlation coefficient is denoted in the center of each scatter plot. [file 1756-8935-7-3-S19.zip › 13072_6071034771062435_MOESM19_ESM/6071034771062435_add19/Figure S18c.tif]

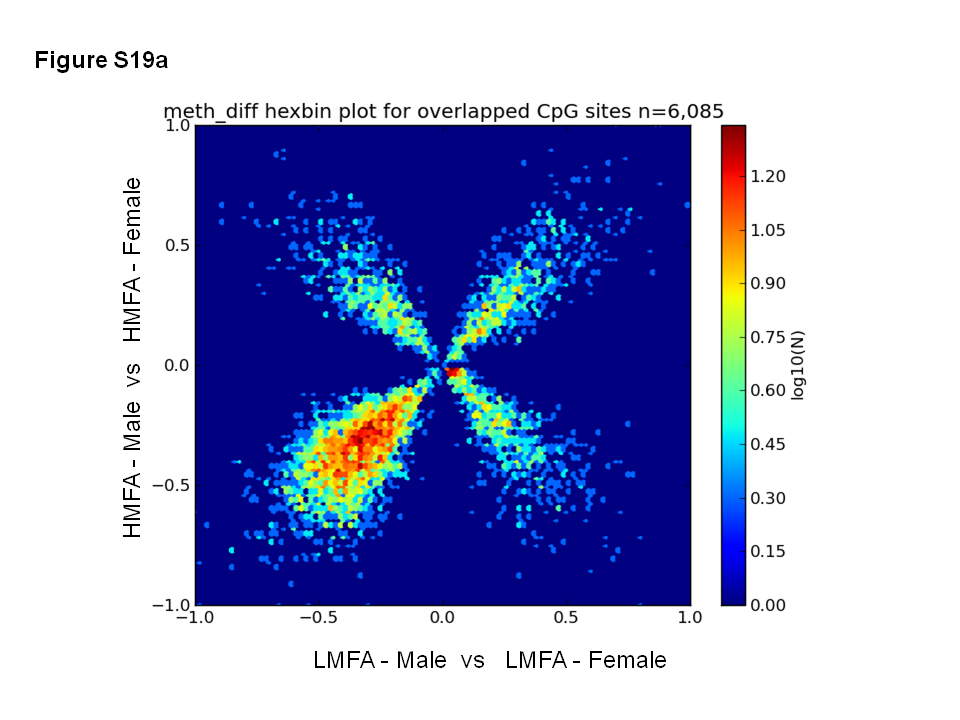

Supplement: Additional file 20: Figures S19 (a, b, c) — Hexbin plot representing the overlapped sites in CpG (n = 6,085), CHG (n = 96), and CHH (n = 154) regions between male and female pups from LMFA compared with high maternal folic acid (HMFA) pups from total significant (P <0.05) differential methylation sites. Each dot in hexbin plot is one of the overlapped sites. The colors blue, green, yellow, and red represent the dot density from lower to higher order in accordance to the prevalence of the overlapping sites. [file 1756-8935-7-3-S20.zip › 13072_6071034771062435_add20/6071034771062435_figs19a.tiff]

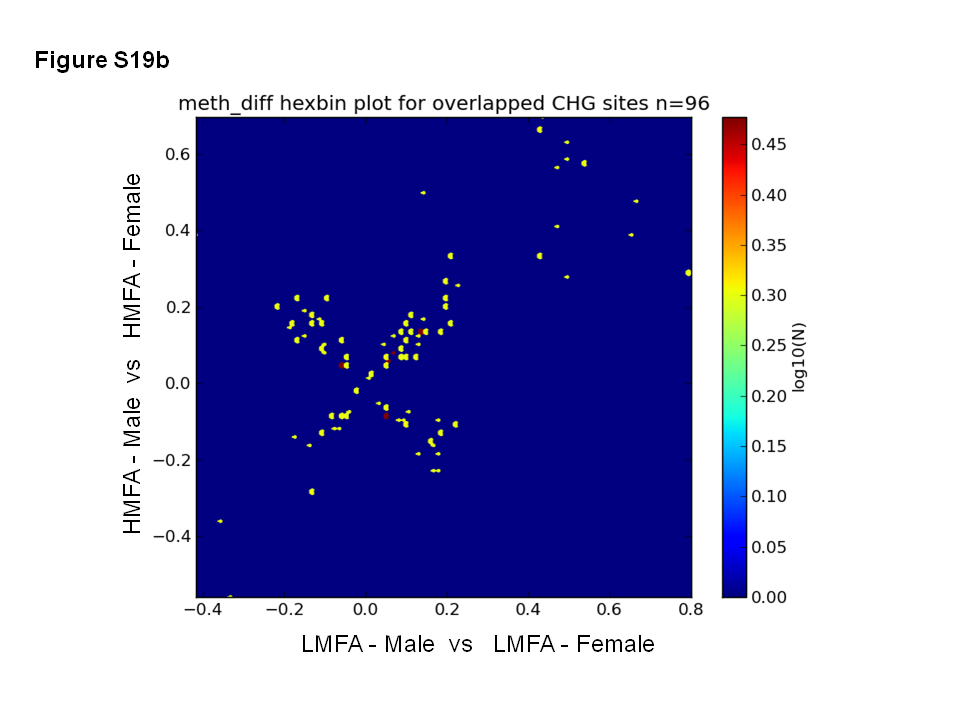

Supplement: Additional file 20: Figures S19 (a, b, c) — Hexbin plot representing the overlapped sites in CpG (n = 6,085), CHG (n = 96), and CHH (n = 154) regions between male and female pups from LMFA compared with high maternal folic acid (HMFA) pups from total significant (P <0.05) differential methylation sites. Each dot in hexbin plot is one of the overlapped sites. The colors blue, green, yellow, and red represent the dot density from lower to higher order in accordance to the prevalence of the overlapping sites. [file 1756-8935-7-3-S20.zip › 13072_6071034771062435_add20/6071034771062435_figs19b.tiff]

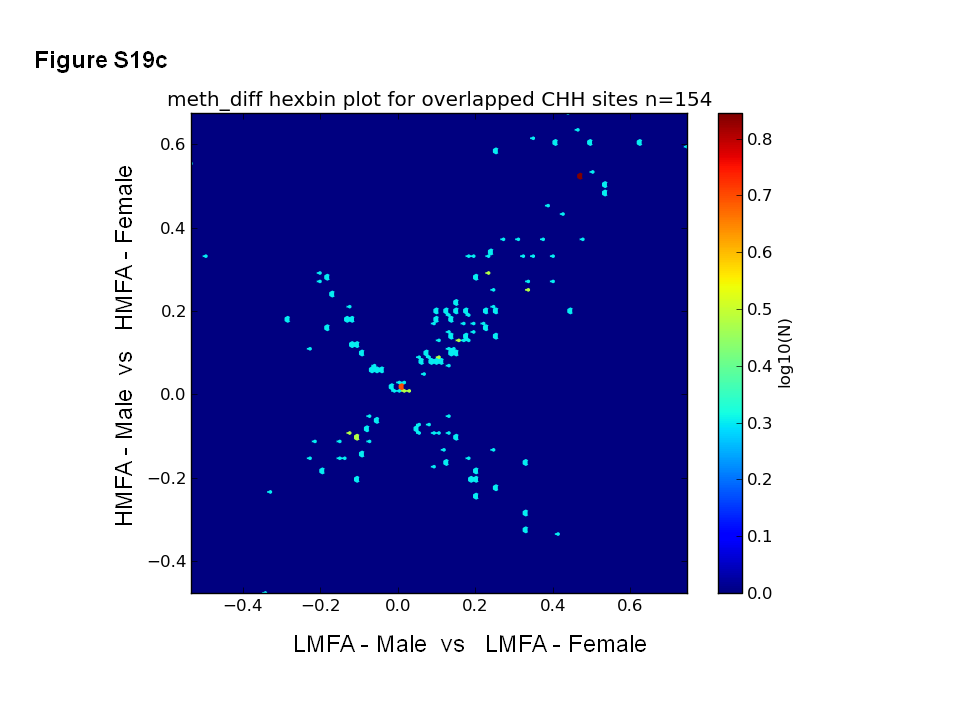

Supplement: Additional file 20: Figures S19 (a, b, c) — Hexbin plot representing the overlapped sites in CpG (n = 6,085), CHG (n = 96), and CHH (n = 154) regions between male and female pups from LMFA compared with high maternal folic acid (HMFA) pups from total significant (P <0.05) differential methylation sites. Each dot in hexbin plot is one of the overlapped sites. The colors blue, green, yellow, and red represent the dot density from lower to higher order in accordance to the prevalence of the overlapping sites. [file 1756-8935-7-3-S20.zip › 13072_6071034771062435_add20/6071034771062435_figs19c.tiff]

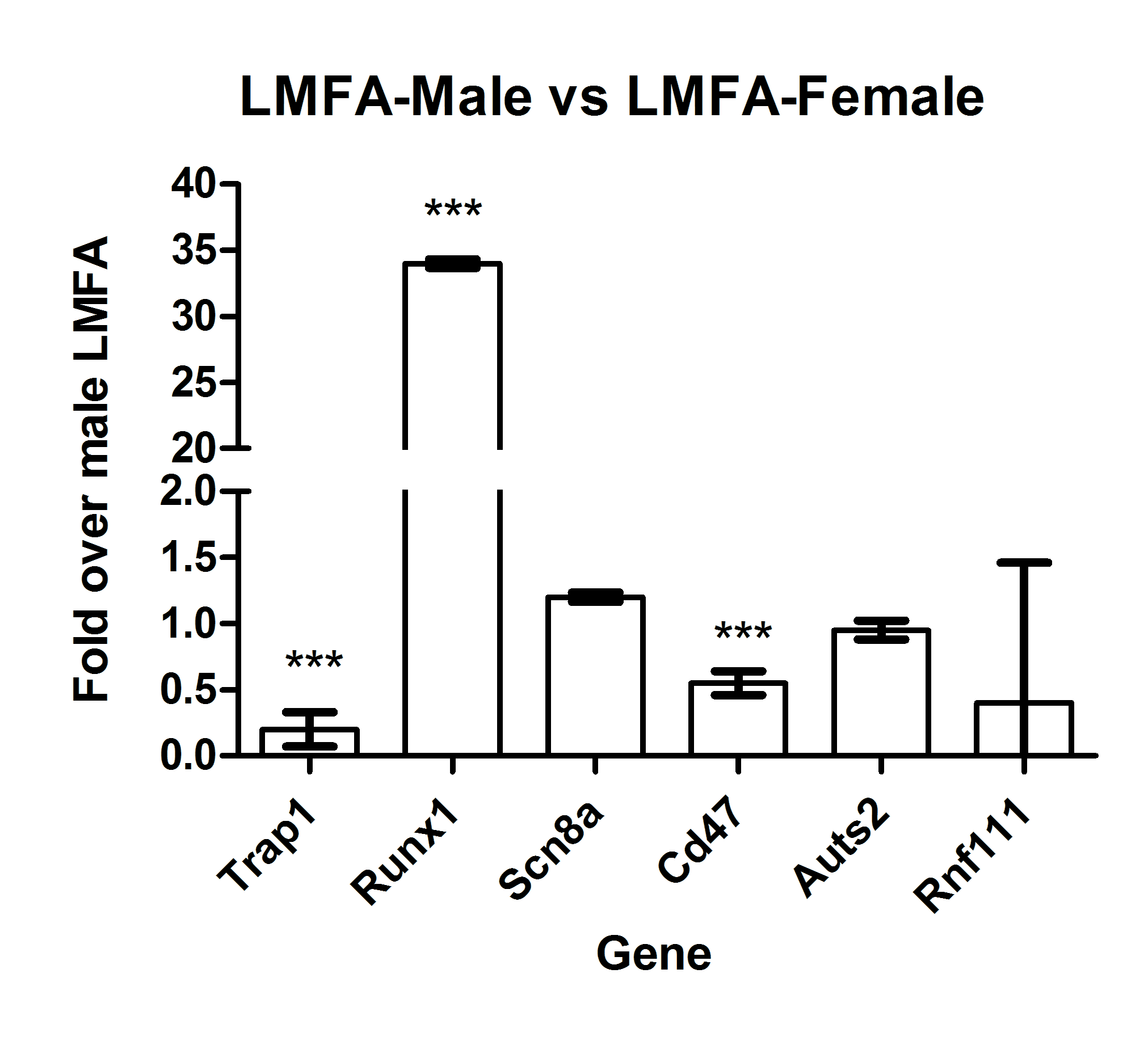

Supplement: Additional file 21: Figure S20a — Quantitative real time reverse transcription-polymerase chain reaction (qRT-PCR ) showing relative expression of the transcripts of genes in female pups that exhibited hypermethylation or hypomethyaltion in the cerebral hemispheres in comparison with male pups from low maternal folic acid (LMFA). The results were normalized to Hprt transcript expression and were expressed as relative values in comparison with corresponding transcripts from male LMFA. Results represent mean ± standard deviation (SD); asterisks denote statistically significant change (*P <0.05, **P <0.01, ***P <0.001). Figure S20b. qRT-PCR showing relative expression of the transcripts of genes in female pups that exhibited hypermethylation or hypomethyaltion in the cerebral hemispheres in comparison with male pups from high maternal folic acid (HMFA). The results were normalized to Hprt transcript expression and were expressed as relative values in comparison with corresponding transcripts from male HMFA. Results represent mean ± SD; asterisks denote statistically significant change (*P <0.05, **P <0.01, ***P <0.001). [file 1756-8935-7-3-S21.zip › 13072_6071034771062435_MOESM21_ESM/6071034771062435_add21/Figure S20a.tif]

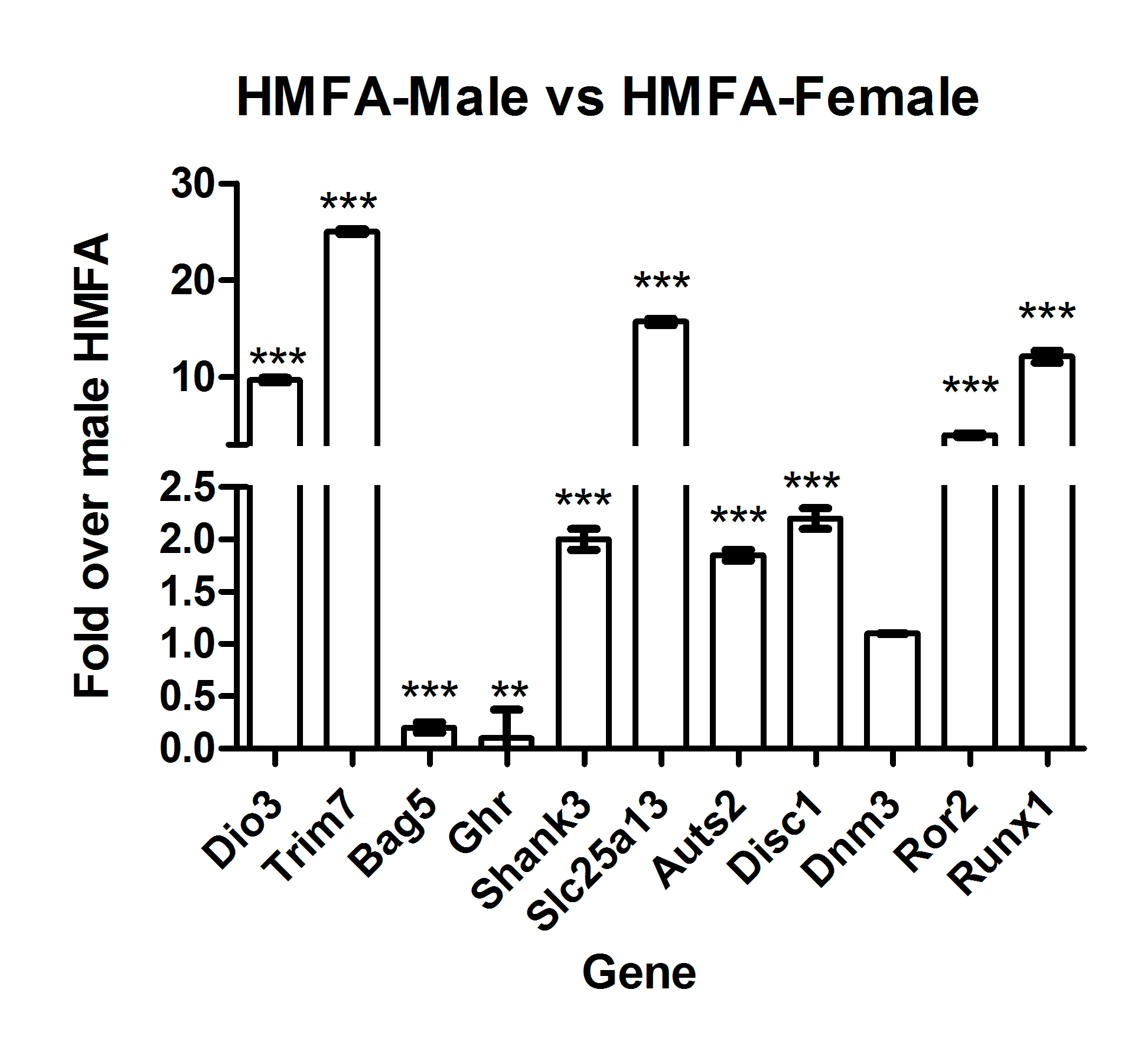

Supplement: Additional file 21: Figure S20a — Quantitative real time reverse transcription-polymerase chain reaction (qRT-PCR ) showing relative expression of the transcripts of genes in female pups that exhibited hypermethylation or hypomethyaltion in the cerebral hemispheres in comparison with male pups from low maternal folic acid (LMFA). The results were normalized to Hprt transcript expression and were expressed as relative values in comparison with corresponding transcripts from male LMFA. Results represent mean ± standard deviation (SD); asterisks denote statistically significant change (*P <0.05, **P <0.01, ***P <0.001). Figure S20b. qRT-PCR showing relative expression of the transcripts of genes in female pups that exhibited hypermethylation or hypomethyaltion in the cerebral hemispheres in comparison with male pups from high maternal folic acid (HMFA). The results were normalized to Hprt transcript expression and were expressed as relative values in comparison with corresponding transcripts from male HMFA. Results represent mean ± SD; asterisks denote statistically significant change (*P <0.05, **P <0.01, ***P <0.001). [file 1756-8935-7-3-S21.zip › 13072_6071034771062435_MOESM21_ESM/6071034771062435_add21/Figure S20b.tif]

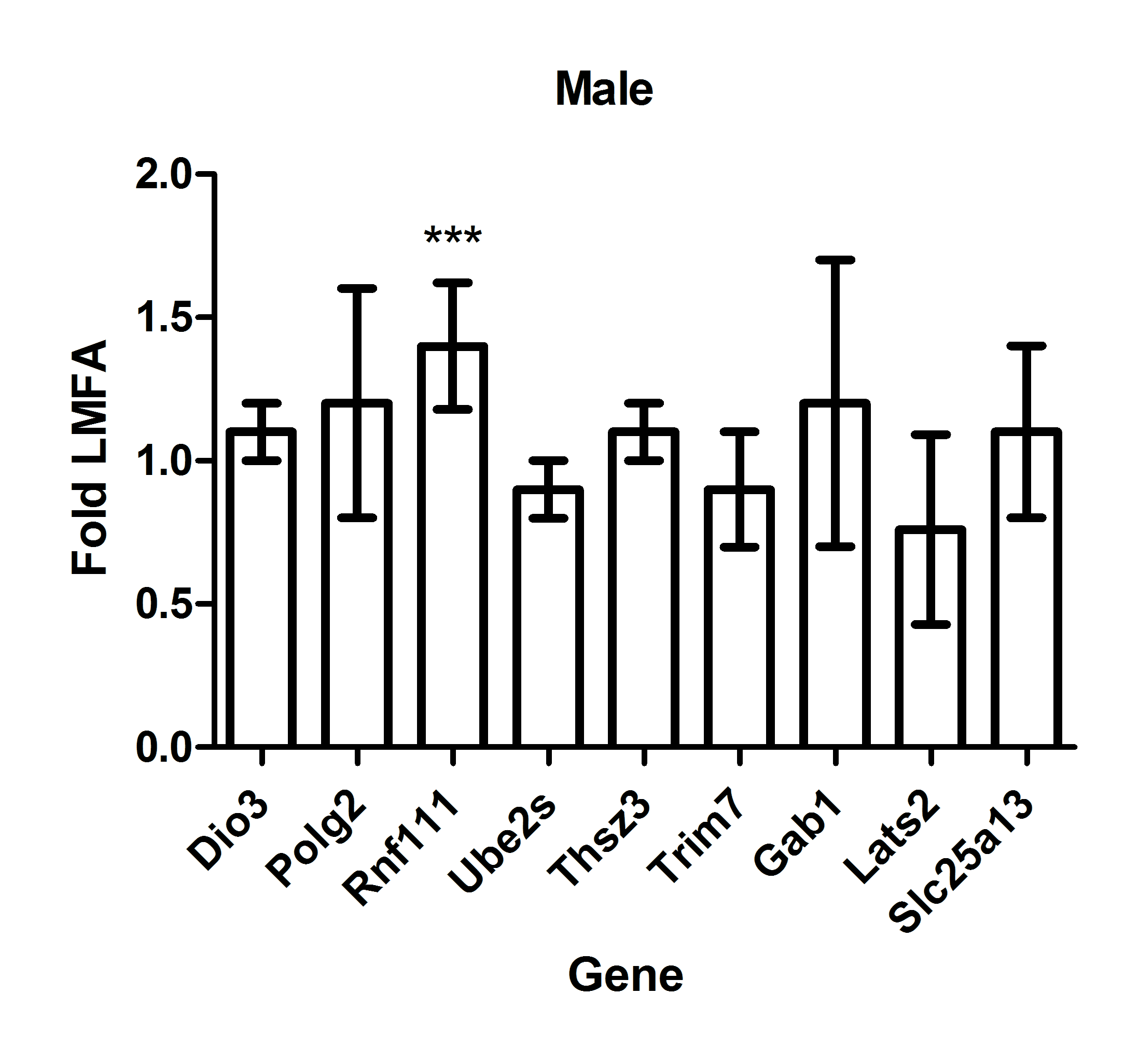

Supplement: Additional file 22: Figure S21a — Quantitative real time reverse transcription-polymerase chain reaction (qRT-PCR) showing relative expression of the transcripts of genes in male pups from high maternal folic acid (HMFA) that exhibited no alterations in the methylation profile in promoter and gene body in the cerebral hemispheres compared with low maternal folic acid (LMFA). The results were normalized to Hprt transcript expression and were expressed as relative values in comparison with corresponding transcripts from LMFA. Results represent mean ± standard deviation (SD); asterisks denote statistically significant change (*P <0.05, **P <0.01, ***P <0.001). Figure S21b. qRT-PCR showing relative expression of the transcripts of genes in female pups from HMFA that exhibited no alterations in the methylation profile in promoter and gene body in the cerebral hemispheres compared with LMFA. The results were normalized to Hprt transcript expression and were expressed as relative values in comparison with corresponding transcripts from LMFA. Results represent mean ± SD; asterisks denote statistically significant change (*P <0.05, **P <0.01, ***P <0.001). [file 1756-8935-7-3-S22.zip › 13072_6071034771062435_MOESM22_ESM/6071034771062435_add22/Figure S21a.tif]

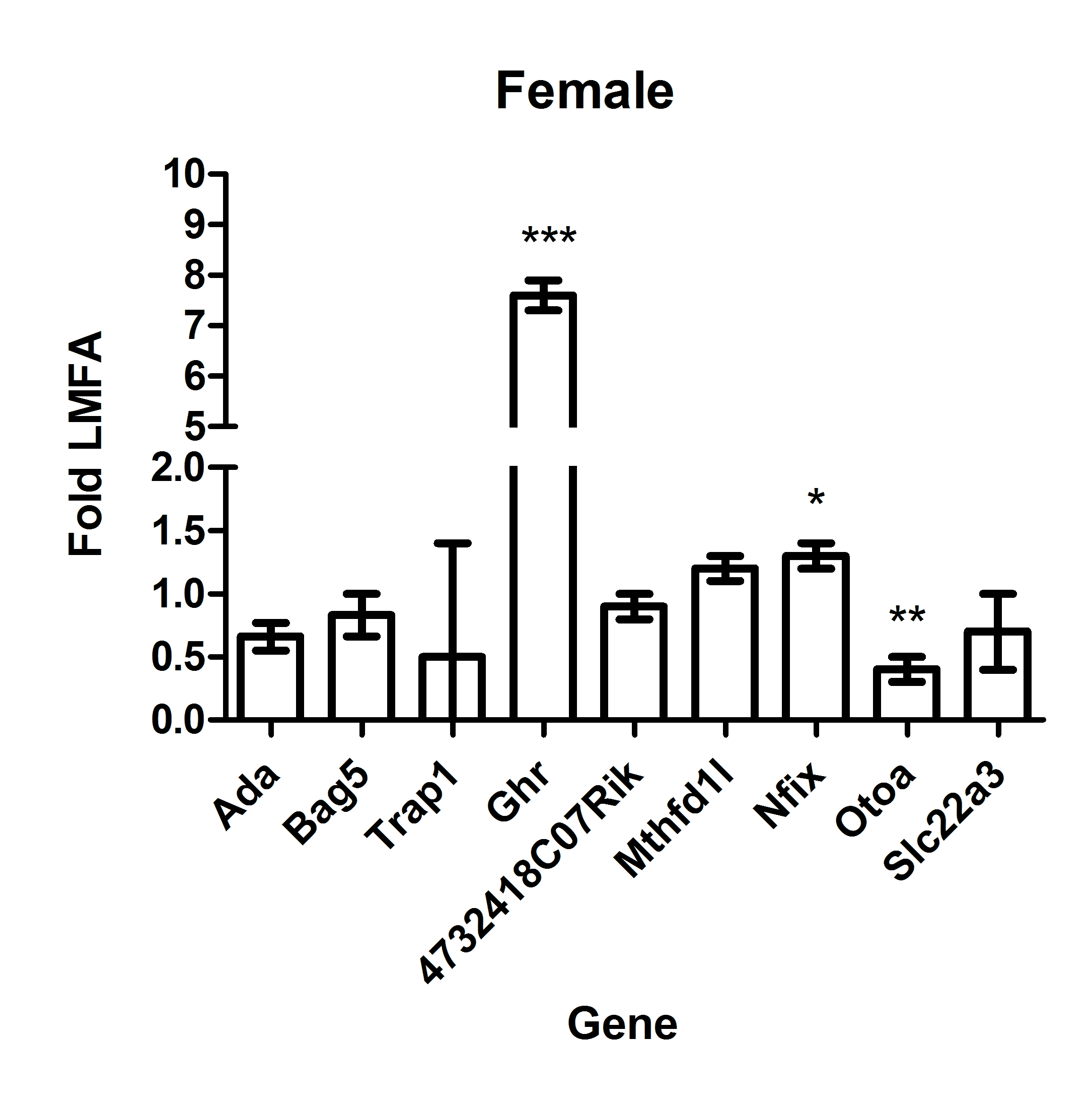

Supplement: Additional file 22: Figure S21a — Quantitative real time reverse transcription-polymerase chain reaction (qRT-PCR) showing relative expression of the transcripts of genes in male pups from high maternal folic acid (HMFA) that exhibited no alterations in the methylation profile in promoter and gene body in the cerebral hemispheres compared with low maternal folic acid (LMFA). The results were normalized to Hprt transcript expression and were expressed as relative values in comparison with corresponding transcripts from LMFA. Results represent mean ± standard deviation (SD); asterisks denote statistically significant change (*P <0.05, **P <0.01, ***P <0.001). Figure S21b. qRT-PCR showing relative expression of the transcripts of genes in female pups from HMFA that exhibited no alterations in the methylation profile in promoter and gene body in the cerebral hemispheres compared with LMFA. The results were normalized to Hprt transcript expression and were expressed as relative values in comparison with corresponding transcripts from LMFA. Results represent mean ± SD; asterisks denote statistically significant change (*P <0.05, **P <0.01, ***P <0.001). [file 1756-8935-7-3-S22.zip › 13072_6071034771062435_MOESM22_ESM/6071034771062435_add22/Figure S21b.tif]
