# Supplementary material for: Single-base resolution of mouse offspring brain methylome reveals epigenome modifications caused by gestational folic acid
Source: Epigenetics Chromatin. 2014 Feb 3;7:3. doi: 10.1186/1756-8935-7-3 (PMC3928622; doi:10.1186/1756-8935-7-3)
Supplement: Additional file 24: Table S12 — List of primers used for quantitative real time reverse transcription-polymerase chain reaction (qRT-PCR) in this study. [file 1756-8935-7-3-S24.pdf]

Table-S12: List of primers that were used for qRT-PCR in this study

**Name**	**Sequences (5' → 3')**
Ada-F	ATGCCTCTCTTCTTGCCAAA
Ada-R	ATGCCTCTCTTCTTGCCAAA
Bag5-F	AATGGAGGGGTGTTGGTTTC
Bag5-R	ACAACGCTGCGGAAGTGTA
Slc5a1-F	CCACAAAGTGACCACTTCCA
Slc5a1-R	GTGGTACCGTTGGAGGCTT
Ghr-F	TTCCAGAAAATGTGCTGCTG
Ghr-R	GAACCCGCGTTCTGTCTC
4732418C07Rik-F	TTCCTGGTAGGTCAGCATCC
4732418C07Rik-R	AAAACCTCTCTCCCTGCCTG
Dio3-F	CCTGAGAGCAAGCCAAAAAC
Dio3-R	ACACAGATGAGCACAGCCAC
Slc22a3-F	AGGAAGCCAAGGTTCAGGAT
Slc22a3-R	CGCTATGTGGAGACCCACTC
Wif1-F	GCATTCTTTGTTGGGCTTTC
Wif1-R	CCATCAGGCTAGAGTGCTCA
Mtnr1b-F	AGGCTATCACCAAGTCAGCC
Mtnr1b-R	GGAACCTGCTTGTCATCCTC
Gab1-F	CCACTGCGCAACACAAAC
Gab1-R	GAAGTGGTTTGCTCGGGAT
Lats2-F	CCAAGGCAGCTTCGATACTC
Lats2-R	GCAGATGCTTCAGGAGTTGG
Runx1-F	GGATCCCAGGTACTGGTAGGA
Runx1-R	GCCATCAAAATCACAGTGGA
Slc25a13F	CACAGGACAGACTCAAAGGCT
Slc25a13-R	CAACCCGAAAACTGTGGAAC
Cd47-F	CCAAACTTTCCCCAGAACAG
Cd47-R	AGGAGGAGAAAGGAGGTTGC
Disc1-F	CACAGTGTTTGCCTTCATGC
Disc1-R	GTGCAGCTCTCTGAGGAGGA
Dnm3-F	GAACCTGACTGCTCCTCTGC
Dnm3-R	CCAGGTCCTGCTACTGATTGA
Evl-F	TCTGGATGTCCATCTCCTCA
Evl-R	TGCTCTTTGCCCTGAACATC
Scn8a-F	ATGTACAAGGCAGGAGTGGC
Scn8a-R	CTGGAGGACTTTGACCCGTA
Homer2-F	GGGTCAATCTGGAAGACGTG

Homer2-R	CGCGTCGACTAGTACGGG
Auts2-F	CAGCACCTCTAGTCGGGAAG
Auts2-R	CTTCCTTGCGTTCCTCTTTG
Pcnxl3-F	CTCATGGTTCAGCCAGGTCT
Pcnxl3-R	GTGTTTGACCTCCGCAAGAT
Hmgb2l1-F	TGGTCAGCCACAATGGTTAC
Hmgb2l1-R	CAGAGACAGAGAGAGGGGAGA
Ror2-F	CTTTCAGAGTGGGAAGTGGG
Ror2-R	CTCCTGCTCTGGACACCCT
Mrps12-F	GAGGGCTAGGCCACGACT
Mrps12-R	CGCTAGGTTGGTGAGGGAC
Ceacam2-F	GCTGAGTCACTGGCTTGTGT
Ceacam2-R	GGTCACCATGAAGGATACGG
Mtap4-F:	ATCCACAAGACTGAGGTCGG
Mtap4-R:	TCCTCTCTGCCCTCTCCC
Shank3-F	CTTTGCATAGCTGGGGGTT
Shank3-R	CCTTCCAGGTGGCCATTATT
Hprt1/Otoa	SA Biosciences (Frederick, MD, currently a Qiagen Co).
